# The sex-dependent response to psychosocial stress and ischaemic heart disease

**DOI:** 10.3389/fcvm.2023.1072042

**Published:** 2023-04-21

**Authors:** Tessa J. Helman, John P. Headrick, Nicolas J. C. Stapelberg, Nady Braidy

**Affiliations:** ^1^ Centre for Healthy Brain Ageing, School of Psychiatry, University of New South Wales, NSW, Sydney, Australia; ^2^ School of Pharmacy and Medical Sciences, Griffith University, Southport, QLD, Australia; ^3^ Faculty of Health Sciences and Medicine, Bond University, Robina, QLD, Australia

**Keywords:** sympathetic nervous system, takotsubo cardiomyopathy, chronic stress, sexual dimophism, glucocortcoids, coronary artery disease

## Abstract

Stress is an important risk factor for modern chronic diseases, with distinct influences in males and females. The sex specificity of the mammalian stress response contributes to the sex-dependent development and impacts of coronary artery disease (CAD). Compared to men, women appear to have greater susceptibility to chronic forms of psychosocial stress, extending beyond an increased incidence of mood disorders to include a 2- to 4-fold higher risk of stress-dependent myocardial infarction in women, and up to 10-fold higher risk of Takotsubo syndrome—a stress-dependent coronary-myocardial disorder most prevalent in post-menopausal women. Sex differences arise at all levels of the stress response: from initial perception of stress to behavioural, cognitive, and affective responses and longer-term disease outcomes. These fundamental differences involve interactions between chromosomal and gonadal determinants, (mal)adaptive epigenetic modulation across the lifespan (particularly in early life), and the extrinsic influences of socio-cultural, economic, and environmental factors. Pre-clinical investigations of biological mechanisms support distinct early life programming and a heightened corticolimbic-noradrenaline-neuroinflammatory reactivity in females vs. males, among implicated determinants of the chronic stress response. Unravelling the intrinsic molecular, cellular and systems biological basis of these differences, and their interactions with external lifestyle/socio-cultural determinants, can guide preventative and therapeutic strategies to better target coronary heart disease in a tailored sex-specific manner.

## 1. Introduction

The mammalian stress response modulates whole body physiology and behaviour to enhance survival in the face of acute environmental threats. Unfortunately, prolonged or repetitive activation of this survival response—*via* a diversity of psychosocial, economic and environmental stressors prevalent in modern society—detrimentally impacts physiology, mood and behaviour to promote major diseases “plaguing” modern populations. These psychosocial stress dependent diseases include major depressive disorder (MDD) (1), obesity, metabolic syndrome and diabetes (2), osteoporosis (3), cancers (4) and cardiovascular disease (CVD) (5). They are also frequently co- or multi-morbid, consistent with these “diseases of modernity” sharing mechanistic networks (6). Modern stress-dependent syndemics are also emerging, with attention to stress and CVD linked synergistic conditions (7, 8), and most recently to stress and the COVID-19 mental health syndemic (9–11).

A primary focus in stress research has been the development of mood disorders, and though the mechanistic basis of stress-dependent MDD remains elusive, these studies provide a (growing) catalogue of the biological influences of chronic stress, including mechanisms likely participating in the positive relationship between stress and CAD. These include changes in nervous and endocrine control of the cardiovascular system, whole body metabolism, immuno-inflammatory function and gut biology, together with affective/behavioural responses that reinforce cardiometabolic disease development (including inactivity, hyperphagia and biased selection of palatable sugar- and fat-rich foods). Critically, the mammalian stress response and its systemic influences are highly sex-dependent, a fundamental yet still under-studied basis for sex specific disease risks and outcomes (12).

## 2. Stress, sex and CAD

Psychosocial and other forms of stress are powerful “unconventional” risk factors for CVD (5). Indeed, animal studies suggest stress may be a stronger determinant of coronary disease than cholesterol and associated lipid levels (13). This pathogenic influence was initially flagged by Selye shortly after his pioneering work defining stress, postulating that chronic stress may be linked to cardiovascular disease (14). Since that time the cardiovascular impacts of stress have been interrogated in pre-clinical, clinical and epidemiological investigations. The latter identify significant influences of diverse stressors on CVD. Psychosocial stress promotes atherosclerosis, CAD and acute myocardial infarction (AMI) (15–17). The INTERHEART study, for example, found risk of AMI was more than 2-fold higher in people reporting “permanent stress” (18). Both personal and work stressors have been linked to up to a 50% increase in CAD (5). The Stockholm Heart Program (SHEEP study group) found AMI patients were more likely to have high level work stress (high work demands vs. low control) (19). The Whitehall II study of British civil servants revealed a >2-fold increase in CVD risk in men experiencing a mismatch between work effort and reward (20), and 1.4-fold increased CAD risk in both men and women experiencing job insecurity (21). Analysis of Jackson Heart Study data indicates a 2.4-fold increased risk of CAD with medium to high level financial stress (an association largely explained by depression, together with smoking and diabetes) (22). Non-obstructive forms of coronary disease are also significantly related to psychological stress and a “distressed” personality (23).

Acute episodes of mental stress, more commonly encountered than chronic forms of stress, are also strongly linked to incidence of cardiovascular events (24, 25). Indeed, the pathological influences of acute “trigger” stressors can appear stronger than pro-disease effects of chronic stress (24). For example, earthquake has been linked to up to a 5-fold increase in sudden cardiac death (26). Acute emotional upset is frequently reported by patients in the hrs immediately prior to AMI (27, 28). Analysis of INTERHEART data suggests emotional upset increases the risk of AMI almost 2.5-fold (27), while meta-analysis supports an almost 5-fold increase in risk of infarction or acute coronary syndrome (ACS) within 2 h of an acute anger episode (29). While still incompletely understood, the pathophysiology of spontaneous coronary artery dissection (SCAD)—an important determinant of AMI and sudden death—may also involve acute psychosocial or physical stress (30, 31).

The associations between stress and chronic disease development are strongly sex-dependent, however the importance of sex in these linkages remains understudied. Recognising the need to address this historic paucity of research, investigators have belatedly focussed attention on sex differences (32), including in stress biology and its influence on cardiovascular or mood disorder development (33). Although females may be conferred some protection against CAD development, this is evident prior to menopause (34), whereas post-menopausal women may be at greater risk of AMI than men and suffer greater morbidity and mortality (35). Both short- (36) and long-term outcomes (37) appear worsened in women, though these differences may reflect in part higher age and co-morbidities (38) and are minimised in high-quality clinical settings (39). Nonetheless, considerable evidence indicates that short- and long- term outcomes in acute coronary syndromes are consistently worse in young to middle-aged women relative to age-matched men (40, 41). A greater prevalence of anxiety and depression in women also reduces adherence to cardiac rehabilitation (42), contributing to worsened long-term outcomes. Sex differences in stress biology contribute to these biased outcomes. Indeed, while psychological stress significantly predicts coronary events in women, this is less evident in men (17). Women are at particularly heightened risk of stress-dependent SCAD (30, 31) and myocardial infarction (43,44), together with Takotsubo syndrome—a coronary-myocardial disorder induced by profound stress (45, 46). A higher female prevalence of heart failure with preserved ejection fraction (HfpEF) adds further support to the importance of sex in coronary related disorders, given the involvement of microvascular dysfunction and inflammation in this condition (47).

In this review we briefly outline the mammalian stress response and consider how it differs fundamentally between the sexes, before discussing the mechanistic basis of psychosocial stress-dependent heart disease and evidence these processes are also highly sex-dependent.

## 3. The stress response: distinct in males and females

### 3.1. The mammalian stress response

**Figure 1:**
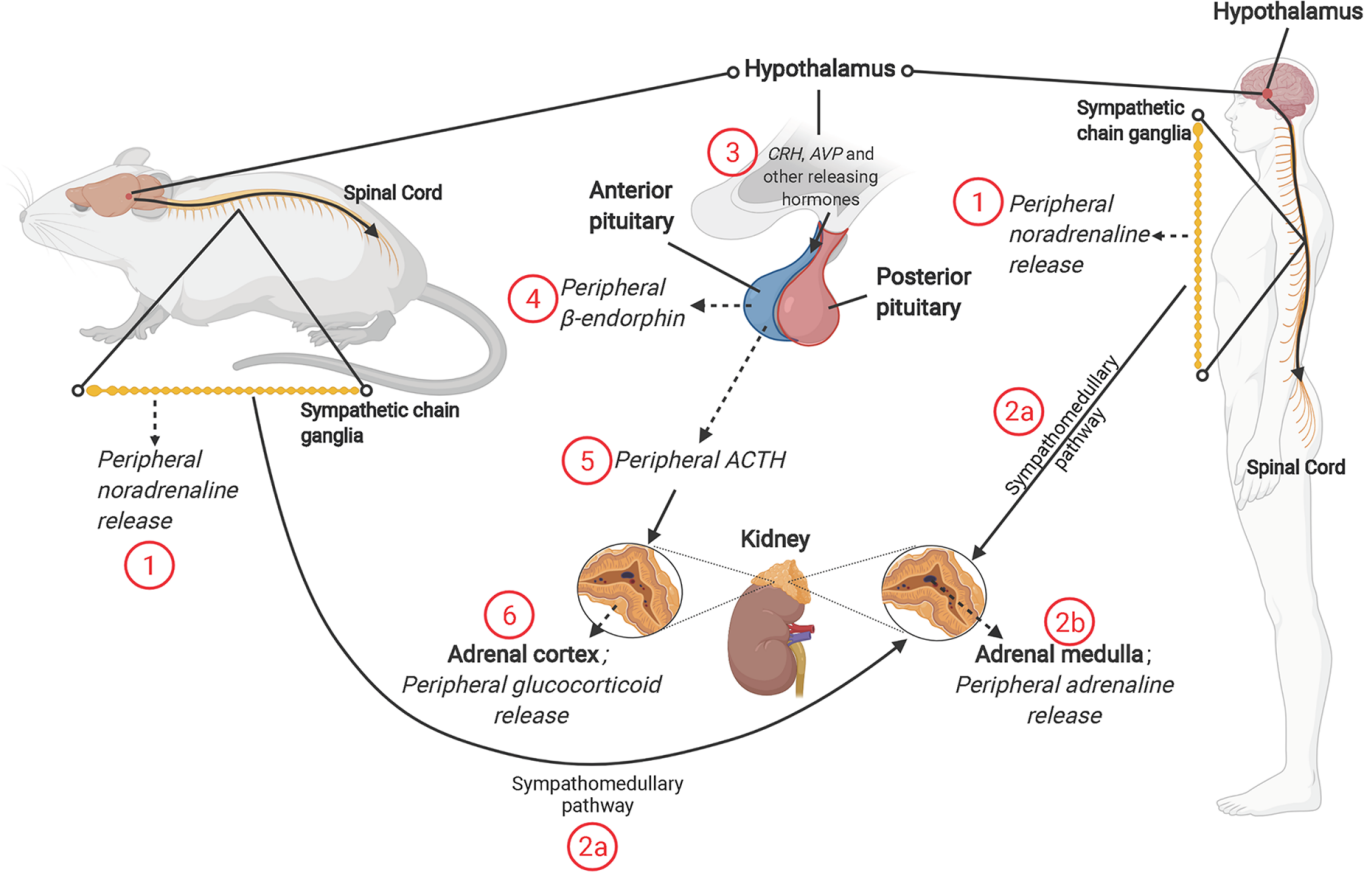
The mammalian stress response, and roles of sympathetic nervous system (SNS) and hypothalamic-pituitary-adrenal (HPA) axis. The SNS releases (1) noradrenaline from the splanchnic nerve (originating from sympathetic chain ganglia) and (2b) Adrenaline from the adrenal medulla [through activation of the sympathomedullary pathway (2a)]. Activation of the HPA axis involves release of corticotropin-releasing hormone (CRH) and other “releasing” hormones [including arginine vasopressin (AVP)] from paraventricular nucleus of hypothalamus (3). The release of CRH, AVP and other hormones acts on the anterior pituitary, resulting in release of β-endorphin (4) and adrenocorticotropin-releasing hormone (ACTH) (5). ACTH acts on the adrenal cortex to promote synthesis and release of glucocorticoid hormones; cortisol in humans and corticosterone in rodents (6). NB: solid line with arrow = act on; dashed line with arrow = release of; solid line = description; solid line with circle = zoom in.

Physical and psychological stimuli threatening or perceived to threaten wellbeing (stressors) induce a systemic stress response—a centrally controlled, integrated adaptation aimed at maintaining homeostasis and enhancing survival (Figure 1). This well conserved mammalian stress response involves 2 primary central mediators—the hypothalamic-pituitary-adrenal (HPA) axis and the autonomic nervous system (ANS). The renin-angiotensin-aldosterone system (RAAS) is also involved, linking stress to regulation of blood pressure and volume.

The paraventricular nucleus (PVN) of the hypothalamus is the first brain region to respond to a real or perceived stressor, in turn promoting HPA axis and sympathetic nervous system (SNS) activities (Figure 1). Release of noradrenaline from peripheral nerves and adrenaline from the adrenal medulla is coupled with a decline in parasympathetic nervous system (PNS) activity, contributing to an autonomic imbalance. While normally tightly controlled, prolonged or chronic stress and HPA activation stimulates additional brain regions, including the amygdala and medulla oblongata, and leads to a hyper-stimulation of the PVN and the HPA axis. Hypothalamic neurons are stimulated to synthesize corticotropin releasing hormone (CRH) and arginine vasopressin (AVP). At the hypothalamic-pituitary unit of the axis, CRH is released into the hypophyseal portal system, with adrenocorticotropic hormone (ACTH) cleaved from its pro-opiomelanocortin precursor, simultaneously generating β-endorphin. This surge in ACTH stimulates adrenal cortical cells to release glucocorticoids and adrenal androgens (Figure 1). The primary glucocorticoid is species dependent—cortisol in humans and corticosterone in rodents—though functions and activities are similar.

Importantly, the initial perception of stress, activation of these stress pathways and their subsequent influences on physiology and behaviour appear to be highly sex-dependent. Transcriptomic profiling of brain responses to stress is informative regarding the extent of these differences (48, 49), which appear to be of a similar magnitude to the differences between rodent and human biology (50). Disentangling the integrated elements underlying the sex-specific stress response remains a major challenge. Sex differences involve a complex interplay between proximate biological mechanisms, including chromosomal and gonadal determinants of nervous system structure and function (51–53) that act developmentally and post-developmentally (54), molecular transduction of life history *via* epigenetic control across the lifespan (particularly in early life) (55, 56), and the influences of (sex-biased) socio-cultural (57), economic and environmental factors. Women may generally experience higher degrees of background stress in day-to-day life, for example as a result of ongoing inequities in unpaid domestic work and other stressors (58–61). In addition, social support and ranking substantially influence stress resilience and reactivity, however such effects differ considerably between the sexes (62, 63). Indeed, as argued by Cohen et al. (57), social construction of gender roles may largely explain sex differences in psychosocial stress reactivity.

Nonetheless, the mammalian stress response itself appears to be characterised by sex-specific features across organisational levels (64): from the initial perception and processing of stressors; to differing fear, cognitive and coping responses; functionality and reactivity of integrated HPA axis, sympatho-adrenergic and inflammatory pathways (including the locus coeruleus-noradrenaline-neuroinflammatory axis); neurotrophin signalling and neuroplasticity; and signalling *via* multiple endocrine and neurotransmitter systems. The gut microbiome, the gut-brain axis and its involvement in stress-related disease, also appear sex-dependent, as are the influences of early life programming/epigenetic control, and the conditioning effects of prior stress. Our own recent work supports greater biological stress or allostatic load in female *vs*. male rodents subjected to chronic social stress, with evidence of greater coronary dysfunction, anxiety-like behaviour, weight loss and inflammation in females (12). However, mixed findings emerge from different animal studies, including evidence females are more susceptible to neuroendocrine and behavioural disruption (and selectively more sensitive to the cardiovascular/autonomic effects of homotypic stress) while males are more vulnerable to somatic effects of chronic stress (65).

### 3.2. Evolutionary perspective

Since advantageous psychological traits differ between males and females during human evolution, Darwinian sexual selection provides a mechanistic basis for the emergence of behavioural/cognitive differences between the sexes (66). This process selects for traits that increase competitiveness in acquiring and fertilising mates. A general pattern is that females, with slower reproduction rates, invest more in parenting and mate selection, and less in intra-sexual competition for mates; while males with a faster reproduction rate invest less in parenting/mate selection, and more in competition. In Palaeolithic life females shared care of infants, food gathering and female-female social interactions, whereas males had differing roles and pressures, including social isolation for extended periods. Females thus invest in developing social support, and attracting supportive mates for reproduction, while males engage in rank behaviour to enhance access to multiple mates, acquire resources and maintain social status (67). Intra-sexual competition for mates favours aggression/territoriality. Sex-dependent responses to prenatal stress may also have an evolutionary origin: it may be adaptive for females to be more alert to dangers, thus more stress responsive (potentially predisposing to stress-related disorders) (68). As detailed below, males and females differ substantially in their initial perceptions of stress, stress reactivities, and cognitive and coping responses.

### 3.3. Sex influences stress perception, reactivity, and cognitive responses

Differing perception of stress, and heightened reactivity, arousal and negative valence may contribute to the sex-dependence of stress-related disease development. Distinct cognitive and coping responses influence stress resilience and systemic outcomes.

#### 3.3.1. Stress perception and hyperarousal

What is perceived as a threat or stressor can be highly diverse at an individual level, influenced by extrinsic environmental (social, cultural, economic) and intrinsic (chromosomal, gonadal) determinants, together with the epigenetic transduction of life history and experience. Evidence has shown that females have a generally greater perception of stress [and also pain (69)], which may influence disease processes (70–73). Interestingly, while perceived stress and coping have been linked to stress-inflammatory function, there is also evidence of an inverse relationship in Japanese men (74, 75). The differing stress perception may contribute to hyperarousal, a maladaptive state leading to agitation, restlessness and cognitive disruption. A core feature of stress-related psychiatric disorders, hyperarousal is more pronounced in women than men (76, 77).

#### 3.3.2. Fear conditioning and extinction

Fear conditioning and extinction, important in mitigating effects of repeated stressors, is mediated in animals and humans by circuitry linking prefrontal cortex and amygdala (78, 79). Fear responses and these neuronal circuits differ between the sexes (80–82), which may reflect differing neurogenesis responses (83), and predispose to a heightened stress reactivity in females.

#### 3.3.3. Cognitive responses

Cognitive responses to stress influence outcomes, with evidence improved cognitive function reduces inflammatory reactivity. For example, Shields et al. (84), shows better cognitive control reduces salivary cytokine levels in response to a video stressor. Sex significantly influences cognitive strategies (85). Cognitive responses to stress differ between the sexes, involving in part gonadal hormone differences (86–88). There is evidence stress may impair decision making to a greater extent in females than males (89).

### 3.4. Biological basis of sex-dependent stress responses

Determinants of sex-dependent stress responses encompass sex-specific genetic interactions (90), sex hormones (91) and associated endocrine changes (92). Animal studies confirm the importance of both gonadal and genetic sex (90), for example employing the four-core genotype mouse model (95, 96). Sex also oppositely affects gene modules related to stress (96), a finding also evidenced in humans (97). Immuno-inflammatory reactivity differs, with female-specific changes in peripheral (93) and central inflammation (94), and links between inflammation and behaviour (93, 98). Overall, differing behavioural and physiological influences of stress may be explained by sex differences in brain architecture and circuitry, the function and reactivity of the HPA (99, 100) and a locus coeruleus–noradrenergic-neuroinflammatory axis (101), together with neurotrophin signalling and the influences of early life experience and adversity (among other factors).

#### 3.4.1. Brain structure and circuitry

Recent neuroimaging studies support sexual dimorphisms in brain structure. The most agreed-upon difference is the macroscopic observation of a larger brain volume in men (102, 103), even after accounting for body size difference (103–105). Differences are also evident with respect to cortical and sub-cortical regions, including larger planum temporale and Sylvian fissure in males (106, 107), whereas a larger hippocampus, superior temporal cortex, Broca's area and caudate are observed in females (108, 109). Women also appear to have a proportionally higher grey:white matter ratio than men (110, 111). Relevant region-specific differences are also evident. For example, higher grey matter percentages or grey:white ratios are evident within the dorsolateral prefrontal cortex (112), superior temporal gyrus (112), and parietal lobe (104, 113) of the female brain. How such architecture differences might influence the stress response is unclear, however there is evidence linking stress resilience to neuro-anatomy, for example differences in limbic region structure and connectivity (114–116). A reduced hippocampal volume is also characteristic of stress-related depression in both men and women (117, 118).

Differences within specific neural circuits underlying emotional control and expression may be important: regions associated with negative valence, including the amygdala, frontal cortex and hippocampus, exhibit significant sex differences (119, 120). The magnitude of corticolimbic responses to emotional stimuli may also differ between sexes. Domes et al. (121) found that initial aversive stimuli increased amygdala activity to a greater extent in women than men. Stevens et al. (122) also observed sex differences in the amygdala response to emotional stimuli, with women favouring negative and men positive emotion. Negatively valanced words activate left perirhinal cortex and hippocampus in women and the right supramarginal gyrus in men (123). Garret et al. (124) found that chronic stress induces opposing dendritic atrophy and hypertrophy in the frontal cortex of male and female rats, respectively. Stressed females also exhibit longer and more complex dendrites in basolateral projecting neurons (125), an outcome not evident in males (126). These regional activity and circuitry differences contribute to sex specific stress axis activity and responses.

#### 3.4.2. Differences in stress axis and neuroinflammatory reactivities

Prominent sex differences emerge in the HPA axis response to stress (127). In healthy participants, baseline cortisol levels may be comparable in women and men (128, 129), although this is challenged by recent reports of higher baseline concentrations in women (130, 131). This may be related in part to higher background levels of stress in women (59–61). Women with stress-related MDD also have higher cortisol levels than male patients (133). Rodent studies support both higher basal and stress-induced corticosterone levels in females vs. males (134–136), with females secreting higher corticosterone in response to either physical or psychological stressors (135, 137–148). Enhanced corticosterone responses in females involve both a more rapid elevation in the initial minutes of stress exposure (143, 149, 150), and a more sustained elevation in corticosterone levels (135, 149, 151). Sex differences in the corticosterone response are paralleled by higher stress-dependent ACTH levels in females (134, 144, 145, 149, 152, 153).

Differing glucocorticoid responses suggest generally increased stress axis reactivity in females (154, 155). This is also reflected in evidence of increased neuroinflammatory reactivity in females (156, 157). However, the simplistic notion that increased corticosterone/cortisol levels may underpin sex differences in behavioural outcomes and resilience must be tempered by evidence females may be more resistant to the effects of corticosterone (158–160). Moreover, the biological response to cortisol or corticosterone is itself highly sex-dependent, including distinct behavioural, neuroendocrine and miRNA responses (160, 161). Although corticosterone levels predict stress-dependent behavioural changes in males, this may not be the case in females (162).

Multiple mechanisms could contribute to differing HPA axis responses in females vs. males. Sex hormones influence HPA axis function (99, 154, 163–165), including up-regulation of hypothalamic CRH, which has oestrogen and androgen response elements in its genetic promoter region. There is also evidence of an interaction between oestrogen and corticosteroid binding globulin in differing sex-dependent stress reactivity (166). Considerable evidence supports a greater reactivity of the locus coeruleus (LC) norepinephrine system in females (101), important in hyperarousal (167–169) and stress-inflammatory activation. The LC of adult female rats is larger and contains more noradrenaline containing neurons than in males (170–172) and may receive greater synaptic input than in male rats (101). Increased LC-noradrenaline axis reactivity in females may involve: gonadal hormone up-regulation of hypothalamic CRH, which has oestrogen and androgen response elements within its gene promoter region; increased noradrenaline content, involving oestrogen-dependent up-regulation of synthesis/down-regulation of degradation (101); differing locus coeruleus neuronal number/ structure and dendritic morphology, promoting reactivity to psychosocial stimuli (173); differing trafficking of the CRH receptor, which may not desensitise as effectively in females (174, 175); and biased CRH signalling *via* CRH may increase HPA reactivity in females, with distinct receptor-effector coupling in each sex (175, 176).

An increased inflammatory reactivity to stress in females may arise as a result heightened LC-noradrenaline axis (coupled with reduced PNS) activity. Inflammatory challenge itself induces distinct behavioural outcomes in females (177–180). However, experimental findings are again equivocal, including evidence: of similar behavioural outcomes (sickness behaviour) in the face of differing levels of neuroinflammation (181); that sex differences in neuroinflammation do not underlie diverging affective-like behaviours; and that differences in inflammatory markers are not linked to differing corticosterone levels (182). There is also evidence for differing pro- and anti-inflammatory influences of other regulatory systems in males vs. females [e.g., cannabinoid receptor signalling (183)], complicating interpretation. Nonetheless, an array of findings provide support for the thesis that differing stress reactivity and disease development in females involves at least in part greater stress-inflammatory activation (184).

#### 3.4.3. Early life programming and life history

Experiential factors significantly influence stress resilience in later life, and these effects may differ between the sexes (185). Early life programming of stress reactivity and associated disease risks are sex-dependent, involving shifts in neuro-immune and other functions (180, 186–190), together with differences in neurogenesis/plasticity (88, 180, 190). There is evidence adverse childhood experience may have a greater impact on adaptive responses to stressors in later life in women vs. men (191). Dimorphic influences of chronic stress in later life may thus involve both prior programming effects and extant sex differences. Sex dependent influences of early life stress and palatable food intake include reportedly select hyperphagia/weight gain in females vs. metabolic programming (adrenal growth and declining adiponectin) in males (192). Prior experience of controllable stress also has a conditioning or pro-resilience effect, protecting against subsequent stress. However, animal studies suggest this benefit is effective in males and not females, potentially reflecting differing neuroplasticity (193). Other recent work indicates that early life influences synaptic organization/excitability in the neonatal amygdala in a sex dependent manner, governing anxiety and fear responses in later life (194, 195).

#### 3.4.4. Neurotrophin signalling and neurogenesis

Differing neurogenesis and plasticity responses are evident in males and females, with both early life (196) and adult (197) stressors. Experimental studies support sex specific changes in neurogenesis with chronic restraint (198, 199) and social stress (197), for example. Among neurotrophins, much attention has focused on brain-derived neurotropic factor (BDNF) in stress-dependent disorders, and there is evidence central nervous system (CNS) BDNF signalling is selectively or more substantially disrupted in stressed females than males (200–203). This dimorphism may involve epigenetic (201) and inflammatory (203) mechanisms. Despite a focus on BDNF in CNS responses and affective outcomes, recent evidence implicates BDNF signalling in thrombosis and CAD (204, 205).

#### 3.4.5. Differing pyrimidine nucleotide metabolism and sirtuin signalling

While relatively poorly studied, sex differences in pyrimidine nucleotide metabolism and related sirtuin signalling may be an important determinant of differing resilience to and outcomes from chronic stress in males and females. Stress responses, mitochondrial energy state and metabolic homeostasis, inflammation, oxidative stress, cell senescence and longevity are all sensitive to pyrimidine nucleotides and sirtuin signalling. Nicotinamide adenine dinucleotide (NAD^+^) is an essential pyrimidine nucleotide that serves as a cofactor for several hundred metabolic enzymes (206). NAD^+^ also plays a key role in regulating 3 major groups of NAD^+^ consuming enzymes: sirtuins, poly-ADP-ribose polymerases (PARPs), and CD38/157 ectoenzymes. Intracellular levels of NAD^+^ are reduced in tissues of physiologically aged animals (207, 208) and humans (209–211). Low NAD^+^ is linked to metabolic and age-related disorders (212), while a reduced NAD^+^/NADH redox state may promote cardiomyopathy (213). Chronic catecholamine stress *in vitro* reduces cellular NAD^+^ levels together with PARP-1 expression and activity (214), and chronic stress dependent behavioural changes in animal models have been linked to a NAD^+^–sirtuin-1 pathway (215). Maternal separation stress also modifies whole body NAD^+^ metabolism, with consistently elevated N-methylnicotinamide excretion consistent with net NAD^+^ catabolism (216). Endoplasmic reticulum stress can also repress central quinolinic acid phosphoribosyl transferase transcription, leading to a build-up of the NMDA receptor agonist and excitotoxin, increased oxidative stress and inflammation, and depletion in NAD^+^ levels, culminating in cell death (217, 218).

Supporting casual roles for NAD^+^ in stress-related disorders, nicotinamide mononucleotide supplementation reduces depressive-like behaviour in association with improved NAD^+^ levels, sirtuin-3 activity and mitochondrial energy metabolism in a model of chronic corticosterone stress (219). Schroeder and colleagues document dysregulation of NAD^+^ generation in the brains of chronic prenatally stressed mice, with NAD^+^ therapy preventing axonal degeneration, cognitive and depressive outcomes (220). The same NAD^+^ stabilising therapy counters aberrant maternal care behaviours arising with chronic gestational stress (221).

The sirtuins are at least partly responsible for the effects of NAD^+^ on responses to stress. Sirtuins have been reported to induce stress resistance in lower organisms and mammals (222). Sirtuin-1 is regulated by NAD^+^-dependent PARP-1 and modulates stress responses and resilience, with effects on inflammation, oxidative stress, mitochondrial genesis and metabolism, endothelial dysfunction, coagulopathy, cell senescence and longevity (223, 224). They also play a cardinal role in improving glucose metabolism and insulin secretion (225, 226) *via* increasing NAD^+^ levels (227). Stress related reductions in NAD^+^ therefore have broad implications for these sirtuin sensitive processes. However, stress also directly modifies sirtuin expression and results in inhibitory post-translational modifications, with these changes linked to neurodegenerative and other age-related diseases (228). Oxidative stress and CAD risk factors such as cigarette smoke are known to post-translationally inhibit sirtuin-1 and enhance degradation.

Support for the important of sirtuin-1 in chronic stress resilience includes a significant association between depression and a SIRT1 gene variant identified in a genome-wide sequencing study within a large population of Chinese women (229). Animal studies reveal reductions in hippocampal sirtuin-1 with chronic stress (230–232) and show that pharmacologic or genetic inhibition of sirtuin-1 increases stress-dependent behavioural changes and dendritic atrophy, while activation blocks these outcomes (231). Sirtuin-1 protection against these chronic stress effects may involve a microglial shift toward the anti-inflammatory M2 phenotype (233). Long-term cognitive impairment in response to chronic unpredictable stress in adolescent mice is also associated with increased cortical expression of the 50 kD vs. 110 kD sirtuin-1 isoforms and respective mRNAs (234). Other investigations identify roles for sirtuin-1 dependent pathways in protecting against stress-dependent behavioural dysfunction (235), while the beneficial CNS and behavioural effects of environmental enrichment (236) and hydrogen sulphide (237) have both been linked to sirtuin-1 dependent signalling in rat models of chronic unpredictable stress. The ability of resveratrol treatment to improve chronic stress resilience and outcomes is also linked to changes in central sirtuin-1 signalling (238). On the other hand, Ferland et al. (239), show select infusion of the sirtuin-1 inhibitor sirtinol into the dentate gyrus prevents chronic stress-dependent changes in stress kinase signalling and histone acetylation, corresponding with improved behavioural outcomes. This agrees with evidence sirtuin-1 inhibition may enhances neuroprotection against stress-related depressive behaviour, inflammation and oxidative stress (246). Regional differences in sirtuin changes are important, and may explain differing outcomes. As opposed to a fall n hippocampal sirtuin-1 and sirtuin-3, chronic stress reportedly increases sirtuin-1 in nucleus accumbens, and both pharmacological sirtuin-1 activation or sirtuin-1 overexpression within this region increases depression- and anxiety-like behaviours, while sirtuin-1 antagonist infusion counters these changes (240).

Chronic stress dependent reductions in hippocampal sirtuin-2 are also relevant, with associated depressive outcomes and impaired neurogenesis countered by hippocampal sirtuin-2 over-expression and mimicked by sirtuin-2 inhibition (241). Zhang et al. report that depressive outcomes in chronically stressed mice are associated with CDK5 mediated phosphorylation of sirtuin-2, and are blocked either by gene deletion of sirtuin-2 or inhibition of this phosphorylation process (242). Depressive-like symptoms in rats, and associated oxidative stress and telomere shortening may also involve inhibition of sirtuin-3 and a resultant decline anti-oxidant enzyme activity (243).

##### 3.4.5.1. Sex effects

The importance of sex in stress-dependent changes in pyrimidine metabolism and sirtuin signalling are not well studied. However, a handful of studies support sex differences in NAD^+^ metabolism, and a number have revealed sex specific aspects of sirtuin signalling and stress responses in different tissues. Schwarzmann et al. (244) recently tested for sex differences in NAD^+^ and the NAD^+^ redox ratio in 91 men and 114 women between 18 and 83 years of age. Despite no significant differences in total plasma NAD^+^, a higher NAD^+^/NADH ratio was evident in women vs. men, a difference that declined with age. Thus, the ratio was higher in adult but not elderly women compared to age-matched men. The authors speculate there may be less cellular NAD^+^ release *via* connexin 43 hemichannels and/or increased NAD^+^ consumption *via* enzymes such as CD38 in men. In contrast, Yang et al. (2022) report higher circulating NAD^+^ levels in men than women in a study of 1,518 participants from the Jidong community, 18 years of age and over (245). Breton et al. (246), found no sex differences in blood NAD^+^ levels in a smaller French study, although there was a trend to an age-dependent decline in NAD^+^ in males and not females. Relationships between NAD^+^, sex hormones, and stress have yet to be explored in detail.

A larger, albeit still limited, body of evidence exists regarding sex and sirtuins, which play a role in differing susceptibilities of males and females to CVD (247) and other disorders. Analysis of human ventricular tissue from young and old subjects shows a sex- and age-dependent decline in cardiac sirtuin-1 and -3 expression in females but not males, consistent with declining mitochondrial antioxidant defences and increasing inflammation in female (not male) hearts (248). This may reflect reductions in oestrogenic induction of sirtuins in females. Sirtuin-1 induces sexually dimorphic effects on depressive behaviours in mice, with deletion in forebrain excitatory neurons inducing a depression-like phenotype specifically in male mice (249). Down-regulation of sirtuin-1 contributes to ovariectomy -induced arterial senescence and atherosclerosis in female apolipoprotein (Apo)E^−/−^ mice, whereas oestrogen or a selective oestrogen receptor modulator up-regulate sirtuin-1 and counter these changes (250). Thus, protection against vascular dysfunction and atherosclerosis in females may involve the activity of an oestrogen/sirtuin-1 axis.

Sex-dependent sirtuin-3 signalling produces distinct effects on energy metabolism, coronary and myocardial diastolic function, and tissue stress resistance. Sirtuin-3 depletion in arcuate pro-opiomelanocortin neurons—critical regulators of metabolism—induces a negative energy balance (decreased body weight and adiposity, increased energy expenditure) specifically in male mice on a normal diet, and not in males fed a high fat diet or females on either regimen (251). Such effects may contribute to differing risks of obesity or weight loss with chronic stress in males and females. Sex-specific involvement of endothelial sirtuin-3 in determining blood pressure, coronary flow reserve and diastolic function in female vs. male mice (252) suggests age- or disease related coronary and diastolic dysfunction in females may involve reductions in this signalling. Sex differences in the stress resistance of other tissues is also related to sex-dependent sirtuin-3 levels and activity, which are increased with estradiol and reduced by testosterone (253).

#### 3.4.6. Additional sex-dependent determinants

While focussing on elements of the stress axis and response, other sex differences likely influence the responses to/impacts of acute and chronic stressors. These include differences in monoamine, gamma-aminobutyric acid (GABA), neuropeptide Y (NPY) and adipokine signalling, together with gut biology.

##### 3.4.6.1. Monoamine signalling

Central serotonin activity may be differentially stress responsive in males and females. For example, social stress reportedly decreases action potential frequency in serotoninergic nerves of males while increasing frequency in females (254). Females also appear sensitised to the behavioural effects of serotonin deficiency (255).

The sensitivity of the dopamine system to acute and repeated stressors may be greater in females compared to males (256). Different models of stress are associated with sex-dependent changes in dopamine signalling: the forced swim test increases dopaminergic activity in prefrontal cortex and hippocampus in males compared to females, while chronic mild stress decreases dopaminergic activity in the prefrontal cortex of females (257).

##### 3.4.6.2. GABA signalling

Differences in GABA signalling may also participate in sex-dependent stress reactivity and resilience. Differing behavioural effects of chronic unpredictable stress in male and female mice have been linked to selective changes in pre-synaptic GABA activity in females (258). Others report greater down-regulation of the GABA neuronal marker somatostatin in women with MDD, that somatostatin and GABA-synthesizing enzymes are sensitive to X-chromosome polymorphisms, and that genetic sex modulates both GABA related gene expression and anxiety behaviours (259). Stress dependent up-regulation of GABA related genes is also evidenced in male but not female rats (260).

##### 3.4.6.3. Neuropeptide Y signalling

Neuropeptide Y, linked to and regulating HPA and LC-noradrenaline axis activities, may play a role in the sex-dependent stress response. Studies in rodents support lower central NPY levels in females, including in specific stress-sensitive regions (261). However, there is little information on the sex dependence of NPY within the LC. Gonadal hormones (and fluctuations in their levels) may be important in this regard, with oestrogen increasing NPY neuronal numbers, and hippocampal NPY transcription and release (262–264). Gene deletion studies indicate NPY has a greater role in anxiety behaviours in males vs. females (265, 266). Moreover, the anxiolytic effect of exogenous NPY is evident in males and not females (267). Stress-dependent regional changes in NPY levels also differ between the sexes, though age is an important factor (261). Interestingly, NPY levels increase with age in women (268, 269) while declining in males (270–272).

##### 3.4.6.4. Adipokines

Sex-dependent roles for adipokines warrant study. Chronic stress may differentially modify ghrelin levels and signalling in male and female mice (273), and there is also evidence social isolation stress preferentially inhibits central leptin signalling in female vs. male rats (274). In contrast, restraint stress selectively increases hypothalamus leptin in females (275). We recently report a fall in circulating leptin in socially stressed male but not female mice (12). Increases in leptin, potentially reflecting emerging leptin-resistance, are linked to detrimental cardio-metabolic outcomes (276, 277), and have been linked to disturbed ANS control and reduced heart rate variability (278). Leptin levels appear to be generally higher in women than in men (279, 280).

Adiponectin levels are also higher in females vs. males (279, 281, 282), though this may reflect differing body compositions (279). Adiponectin levels may decline with chronic stress (283), an effect likely to favour cardiac hypertrophy. Adiponectin was recently shown to limit cardiac sympathetic and myocardial remodelling after infarction in dogs (284). Sex differences in adiponectin responses to stress have yet to be examined in detail. Analysis of adiponectin and PTSD in woman supports a protective role for higher circulating adiponectin (285). Oestradiol may increase adiponectin levels in association with improved mitochondrial biogenesis in skeletal muscle (286), a sex dependent protective path potentially extending to the heart. Oestradiol and testosterone have also been shown to induce opposing stimulatory vs. inhibitory effects on mitochondrial biogenesis and adiponectin levels in white adipose tissue (287).

##### 3.4.6.5. Cell death mechanisms

Differences in cell death will influence the response to chronic forms of stress. Previous studies report sex differences in cell death pathways, for example in response to ischemic stress (288). Although caspase-dependent pathways play a dominant role in the female brain, caspase-independent processes associated with activation of PARPs are likely to play a more important role in the male brain (289–293). This is supported by the finding that gene knockdown of PARP-1 reduces infarction volume in the male brain but increases cell death in PARP-1 knockout females (294). Similarly, neuroprotective effects of an NAD^+^ precursor and endogenous PARP inhibitor are evident in male mice but not in females (294). Sex differences in the neuroprotective response has been previously reported in a murine model for reperfusion injury but not in an embolic clot model (295). It is likely that sex differences exist in the links between cellular energy homeostasis and cell death processes.

##### 3.4.6.6. Gut biology and microbiome

Finally, the gut microbiome and gut-brain axis are key players in stress related diseases including cardiovascular, metabolic and mood disorders (296–298), and are sex-dependent and sensitive to gonadal hormones (299–304). Indeed, an oestrogen-gut microbiome axis is implicated in sex and oestrogen sensitive chronic disease (305).

## 4. Sex-dependent stress responses broadly promote CAD

While a summary only of extensive sex differences within the mammalian stress response, it is clear from this overview that the biology of stress (and its influences on health and disease) must be viewed as distinct in males and females. Focussed research is thus essential in identifying and understanding these mechanistic processes within each sex. A historic view of cardioprotection in women *vs*. men belies evidence of worsened CVD outcomes in women (306–308), and a relative increase in CVD risk in females over recent decades *vs*. reductions in males (309). Younger women have a particularly poor prognosis in ACS (310). Stress-dependent disorders, including obstructive and non-obstructive CAD and myocardial infarction (30, 31, 43, 44, 311, 312) and Takotsubo cardiomyopathy (45, 46, 313, 314), are highly sex-dependent, consistent with distinct stress biology. Acute and chronic stressors may influence CAD development and coronary events *via* multiple sex sensitive mechanisms. However, much remains to be unravelled regarding the relationships between sex, stress and CAD.

### 4.1. Acute stress—a powerful trigger

While sustained or repetitive stress promotes the development of chronic diseases including CAD, acute mental stress is also strongly linked to cardiovascular events (24, 25), acting as a potent trigger (24, 26, 315). This acute response may involve shifts in vascular structure and control, inflammation and thrombosis. Acute stress increases vasoconstrictor activity and sympathetic tone (316, 317). An almost instantaneous elevation in cardiac loads (heart rate, arterial pressure) with acute stressors (318) will simultaneously increase ischaemic vulnerability. Episodes of emotional upset or anger are a risk factor for SCAD (30, 31), are common in the hours immediately prior to AMI (28, 319), and appear to profoundly increase risk of acute coronary events (29). Autonomic changes during panic attacks have been linked to significant defects in myocardial perfusion (59), and sympathetic activation is implicated in SCAD (30, 31). Experimentally, acute mental stress induces features of AMI in hypercholesterolemic ApoE^−/−^ mice (60), an effect initially linked to endothelin signalling. However, more recent work supports acute stress-dependent plaque destabilisation (61), mechanistically linked to local noradrenaline activation of endothelial adhesion molecule expression and chemokine release.

Acute stress can increase platelet reactivity, coagulation, and fibrinolytic processes to ultimately enhance thrombosis (320). Platelet activation increases with acute stress (321–323), though there is also evidence platelet glycoprotein (GP)Ib and GPIIb/IIIa expression and function are insensitive to acute stress in healthy subjects (324, 325). These effects appear to be more robust in those with CAD (325, 326). Acute stress also induces a relative hyper-activation of the coagulation cascade, with evidence of reduced fibrinolytic activation in CVD patients compared with healthy controls (327–329). In terms of sex differences, acute stress has been shown to selectively enhance FVIIa activity in men (330) *vs*. increased t-PA activity in women (331).

These acute outcomes involve both glucocorticoid and catecholamine signalling. Platelet activation and aggregation are increased with glucocorticoid administration in healthy subjects (332, 333), and acute catecholamine administration also stimulates thrombopoiesis and platelet activity (*via* α2-adrenoceptors) (322, 334). On the other hand, adrenaline and β2-AR agonists may induce a pro-coagulant state (335, 336), and sympathetic activation acts *via* β2-adrencoepotors to induce endothelial release of FVIII, vWF, and t-PA (322, 337). Increased β-adrenoceptor activity with acute stress may contribute to endothelial dysfunction (338, 339), however this role requires further investigation. The impacts of these glucocorticoid and catecholamine mediated effects may be enhanced by acute stress-dependent inflammation and endothelial dysfunction.

Inflammation is rapidly provoked by acute experimental (340, 341) and real-life stressors, including bereavement, natural disasters, and sporting events (342–344), and may promote coronary ischaemia (345). Increased circulating IL-6 parallels fibrin formation and markers of coagulation, for example (346). The inflammatory response to acute stress may also be exaggerated in those with CAD (347). Acute stress appears to induce endothelial dysfunction, though findings from human studies are mixed (348). Some report increased perfusion and flow-mediated dilation in response to acute stress in healthy males and females (349), while others report vasoconstriction and impaired flow-mediated dilation (350–352). The latter inhibitory effect may be eliminated by inhibiting cortisol release (350), consistent with evidence cortisol reduces vascular eNOS signalling (353, 354). This likely involves a suppressive glucocorticoid response element in the eNOS promoter region (355). Cortisol may also increase release of the potent vasoconstrictor endothelin to further reduce vascular conductance (356, 357). Human studies confirm increased endothelin levels in response to acute stress (358, 359), and a reduction in associated endothelial dysfunction with endothelin antagonism (360).

#### 4.1.1. Sex effects

The importance of sex in the cardiovascular impacts of *acute* stress remain relatively understudied. Females are at a substantially heightened risk of acute mental stress related AMI (Table 1). This stress induced ischaemia (361), and associated transient endothelial dysfunction (362), both predict major cardiovascular events in those with CAD. Vaccarino et al. determined a 4-fold higher risk of mental stress induced ischaemia in women ≤50 years of age compared with men of similar age or older (43). More recent work identifies a 2-fold greater risk of mental stress induced myocardial ischaemia in young women vs. men, with a relationship between peripheral vascular function and ischaemia in women only (44). This is congruent with evidence endothelial function and vascular reactivity to mental stress predict major adverse cardiac events in women but not men (363). These observations collectively suggest greater propensity to microvascular dysfunction and ischaemia in women. Women are also at much greater risk of SCAD and resultant perfusion defects, a response linked to acute emotional and (to a lesser extent) physical stress (30, 31).

**Table 1:** Summary of studies assessing sex dependent cardiovascular outcomes and disease risks.

Study	In-text reference	Number of subjects (female: male)	Psychological findings	Risk profile	Cardiovascular findings
Albert CM, McGovern BA, Newell JB, Ruskin JN. Sex differences in cardiac arrest survivors. Circulation. 1996 Mar 15;93 (6):1170–6. doi: 10.1161/01.cir.93.6.1170.	(584)	355 (84: 271)		Predictors of total and cardiac mortality differ between women and men Coronary artery disease an independent predictor of cardiac and total mortality in women, not men LV ejection fraction the strongest independent predictor of mortality in men, not women	*Among cardiac arrest survivors:* Women less likely to have underlying coronary artery disease than men (45% vs. 80%) Women more likely to have other forms of heart disease than men (*P* < 0.0001) LV ejection fraction higher in women vs. men (0.46 ± 0.18 vs. 0.41 ± 0.18, *P* < 0.05) Increased triglycerides (*P* = 0.02) and LDL-cholesterol (*P* = 0.05) in men compared to women Increased HDL-cholesterol in women vs. men (*P* = 0.0002)
Burger IA, Lohmann C, Messerli M, Bengs S, Becker A, Maredziak M, et al. Age- and sex-dependent changes in sympathetic activity of the left ventricular apex assessed by 18F-DOPA PET imaging. PLoS One. 2018 Aug 14;13 (8):e0202302. doi: 10.1371/journal.pone.0202302.	(364)	133 (69: 64)			Cardiac 18F-DOPA uptake significantly higher in women vs. men (*P* < 0.001)
Cenko E, Yoon J, Kedev S, Stankovic G, Vasiljevic Z, Krljanac G, et al. Sex Differences in Outcomes After STEMI: Effect Modification by Treatment Strategy and Age. JAMA Intern Med. (2018) 178 (5):632–39. doi: 10.1001/jamainternmed.2018.0514.	(35)	8,834 (2,657: 6,177)			*Among STEMI patients* 30-day mortality higher for women vs. men (11.6% vs. 6.0%, *P* < 0 001); higher early mortality risk (after adjusting for comorbidities/treatment covariates) in women vs. men under 60 (OR, 1.88; 95% CI, 1.04–3.26; *P* = 0.02).
Chumaeva N, Hintsanen M, Juonala M, Raitakari OT, Keltikangas-Järvinen L. Sex differences in the combined effect of chronic stress with impaired vascular endothelium functioning and the development of early atherosclerosis: the Cardiovascular Risk in Young Finns study. BMC Cardiovasc Disord. 2010 Jul 12;10:34. doi: 10.1186/1471-2261-10-34	(636)	1,721 (1,002: 719)	Women expressed more negative (*P* = 0.02) and less positive (*P* < 0.001) emotion than men		Chronic mental stress had more significant effect on pre-clinical atherosclerosis (carotid arterial compliance) in men vs. women
Dreyer RP, Ranasinghe I, Wang Y, Dharmarajan K, Murugiah K, Nuti SV, et al. Sex Differences in the Rate, Timing, and Principal Diagnoses of 30-Day Readmissions in Younger Patients with Acute Myocardial Infarction. Circulation. (2015) 132 (3):158–66. doi: 10.1161/CIRCULATIONAHA.114.014776.	(39)	42,518 (11,225: 31,293)			*Among young AMI patients* 30-day all-cause readmission higher for women vs. mean (15.5% vs. 9.7%, *P* < 0.0001)
Kumbhani DJ, Shishehbor MH, Willis JM, Karim S, Singh D, Bavry AA, et al. Influence of gender on long-term mortality in patients presenting with non-ST-elevation acute coronary syndromes undergoing percutaneous coronary intervention. Am J Cardiol. (2012) 109 (8):1087–91. doi: 10.1016/j.amjcard.2011.11.044.	(40)	1,874 (697: 1,177)		Women older with higher incidence of co-morbid conditions vs. men.	*Among NSTEMI patients* No overall sex difference in in-hospital (1.4% vs. 1.6%) or long-term (14.6% vs. 15.8%) mortality. However, women <60 years of age had > 2-fold higher long-term mortality vs. men (*P* = 0.007).
Laitinen T, Hartikainen J, Vanninen E, Niskanen L, Geelen G, Länsimies E. Age and gender dependency of baroreflex sensitivity in healthy subjects. J Appl Physiol (1985). 1998 Feb;84 (2):576–83. doi: 10.1152/jappl.1998.84.2.576.	(594)	117 (“approximately equal number of men and women in each group”)			Higher baroreflex sensitivity in men vs. women in “young” and “middle-age” groups; no significant sex difference in “old” group
Martin EA, Tan SL, MacBride LR, Lavi S, Lerman LO, Lerman A. Sex differences in vascular and endothelial responses to acute mental stress. Clin Auton Res. 2008 Dec;18 (6):339–45. doi: 10.1007/s10286-008-0497-5.	(365)	87 (53: 34)		No significant sex differences in cardiovascular disease risk factors	Increased mean arterial pressure in men vs. women (11.4 ± 1.0 vs. 7.9 ± 0.9 mmHg, *P* < 0.001) Increased heart rate in men vs. women (7.60 ± 1.3 vs. 6.14 ± 0.8 beats/min, *P* < 0.005) Increased endothelial-dependent hyperemia index in male vs. female (13.7% vs. −0.47%, *P* = 0.01)
Mommersteeg PM, Arts L, Zijlstra W, Widdershoven JW, Aarnoudse W, Denollet J. Impaired Health Status, Psychological Distress, and Personality in Women and Men With Nonobstructive Coronary Artery Disease: Sex and Gender Differences: The TWIST (Tweesteden Mild Stenosis) Study. Circ Cardiovasc Qual Outcomes. (2017) 10 (2):e003387. doi: 10.1161/CIRCOUTCOMES.116.003387.	(23)		Women with NOCAD report: more psychosocial distress vs. men (no significant sex- group interaction effect); more anxiety, less positive affect (no differences in depressive symptoms, angina, Type D personality vs. men)		
Nickander J, Themudo R, Sigfridsson A, Xue H, Kellman P, Ugander M. Females have higher myocardial perfusion, blood volume and extracellular volume compared to males—an adenosine stress cardiovascular magnetic resonance study. Sci Rep. 2020 Jun 25;10 (1):10380. doi: 10.1038/s41598-020-67196-y.	(577)	41 (21:20)			Higher myocardial blood volume during adenosine stress (not at rest) in women vs. men. Both higher perfusion and extracellular volume at rest and with adenosine stress test in women vs. men
Ouellette ML, Löffler AI, Beller GA, Workman VK, Holland E, Bourque JM. Clinical Characteristics, Sex Differences, and Outcomes in Patients With Normal or Near-Normal Coronary Arteries, Non-Obstructive or Obstructive Coronary Artery Disease. J Am Heart Assoc. 2018 May 2;7 (10):e007965. doi: 10.1161/JAHA.117.007965	(580)	898 (400: 498)		Less clinical CAD risk factors in women vs. men	Higher 10-year atherosclerotic CVD risk in men vs. women (19.9% vs. 13.8%) Higher rate of obstructive stenosis in men vs. women (across all 4 indications (abnormal stress, NSTEMI, chest pain syndrome, heart failure): *women exhibit less obstructive CAD than men*
Pain TE, Jones DA, Rathod KS, Gallagher SM, Knight CJ, Mathur A, et al. Influence of female sex on long-term mortality after acute coronary syndromes treated by percutaneous coronary intervention: a cohort study of 7304 patients. Coron Artery Dis. (2013) 24 (3):183–90. doi: 10.1097/MCA.0b013e32835d75f0.	(37)	7,304 (1,875: 5,429)		Women significantly older, higher rate of diabetes mellitus vs. men.	*Among ACS patients* Long-term mortality higher in women vs. men (all ACS: odds ratio 1.351, *P* < 0.001]. After multivariate adjustment, female sex not independent predictor of mortality in any ACS.
Samad Z, Boyle S, Ersboll M, Vora AN, Zhang Y, Becker RC, et al; REMIT Investigators. Sex differences in platelet reactivity and cardiovascular and psychological response to mental stress in patients with stable ischemic heart disease: insights from the REMIT study. J Am Coll Cardiol. 2014 Oct 21;64 (16):1669–78. doi: 10.1016/j.jacc.2014.04.087.	(640)	310 (56: 254)		Most clinical risk factors similar between men and women (though women more likely to be non-white, living alone, un- married) Higher proportion of men on a statin Higher proportion of women on an anti-platelet agent (other than aspirin)	*Among stable IHD patients:* Baseline: (a) Heightened platelet aggregation responses to serotonin (*P* = 0.007) and adrenaline (*P* = 0.004) in women vs. men Post stress: (a) Greater MSIMI in women vs. men (57% vs. 41%, *P* < 0.04) (b) Higher collagen-stimulated platelet aggregation responses in women vs. men (*P* = 0.04)
Shivpuri S, Gallo LC, Crouse JR, Allison MA. The association between chronic stress type and C-reactive protein in the multi-ethnic study of atherosclerosis: does gender make a difference? J Behav Med. 2012 Feb;35 (1):74–85. doi: 10.1007/s10865-011-9345-5.	(637)	6,583 (3,458: 3,125)		Higher stress related CRP (inflammatory marker) in women vs. men. Chronic sympathetic-caregiving stress associated with increased CRP levels in women (*P* < 0.05) but not men.	* *
Vaccarino V, Sullivan S, Hammadah M, Wilmot K, Al Mheid I, Ramadan R, et al. Mental Stress-Induced-Myocardial Ischemia in Young Patients With Recent Myocardial Infarction: Sex Differences and Mechanisms. Circulation. 2018 Feb 20;137 (8):794–805. doi: 10.1161/CIRCULATIONAHA.117.030849	(44)	Disease: 306 (150:156); Control: 112 (58:54)	More adverse socioeconomic and psychosocial profiles in women vs. men	No sex difference in cardiovascular disease risk factors	*Among young AMI patients:* Rate of mental stress-induced myocardial ischemia (MSIMI) 2-fold higher in women vs. men Ischemia with conventional stress also 2-fold higher in women vs. men Clinical severity lower in women
Vaccarino V, Wilmot K, Al Mheid I, Ramadan R, Pimple P, Shah AJ, et al. Sex Differences in Mental Stress-Induced Myocardial Ischemia in Patients With Coronary Heart Disease. J Am Heart Assoc. 2016 Aug 24;5 (9):e003630. doi: 10.1161/JAHA.116.003630	(43)	686 (191:495)	More adverse psychosocial profile in women vs. men	No discernible sex difference in cardiovascular disease risk factors	*Among stable CAD patients:* Incidence of mental stress-induced myocardial ischemia (MSIMI) almost 4-fold higher in women vs. mean ≤50 years of age
Zachura M, Wilczek K, Janion M, Gąsior M, Gierlotka M, Sadowski M. Long-term outcomes in men and women with ST-segment elevation myocardial infarction and incomplete reperfusion after a primary percutaneous coronary intervention: a 2-year follow-up. Coron Artery Dis. (2019) 30 (3):171–76. doi: 10.1097/MCA.0000000000000703.	(37)	2,694 (948: 1,746)			*Among STEMI patients:* Higher AMI history (15.4 vs. 11.7%, *P* = 0.0073), PCI (10 vs. 6.7%, *P* = 0.0039) and CABG (2.9 vs. 1%, *P* = 0.0016) in men vs. women Tachycardia more frequent in women vs. men (14.5 vs. 10.3%, *P* = 0.0009). Higher risk of rehospitalization in women vs. men

MI, myocardial infarction; PCI, percutaneous coronary intervention; CABG, coronary artery bypass graft; MSIMI—mental stress induced myocardial infarction; ASCVD: atherosclerotic cardiovascular disease; NSTEMI: non-ST segment elevation myocardial infarction.

There is evidence acute stress exerts distinct effects on mediators of coagulation and fibrinolysis in males and females (330, 331). Greater sympathetic activity in the hearts of women (364), particularly in the apical region of the left ventricle, may be relevant to relative myocardial sensitivity to acute stress, and is consistent with the sex dependence of Takotsubo cardiomyopathy and the apical changes in this disorder. The acute coronary response to mental stress differs between sexes: Martin et al. (365) found that reactive hyperaemia with mental stress is lower in women *vs*. men, in association with greater endothelial dysfunction and reduced reactivity. Mehta et al. (366) found that women with coronary vascular dysfunction also exhibit greater peripheral vasoconstriction in response to acute mental stress.

### 4.2. Chronic stress—multipotent driver of cardiovascular disorders

**Figure 2:**
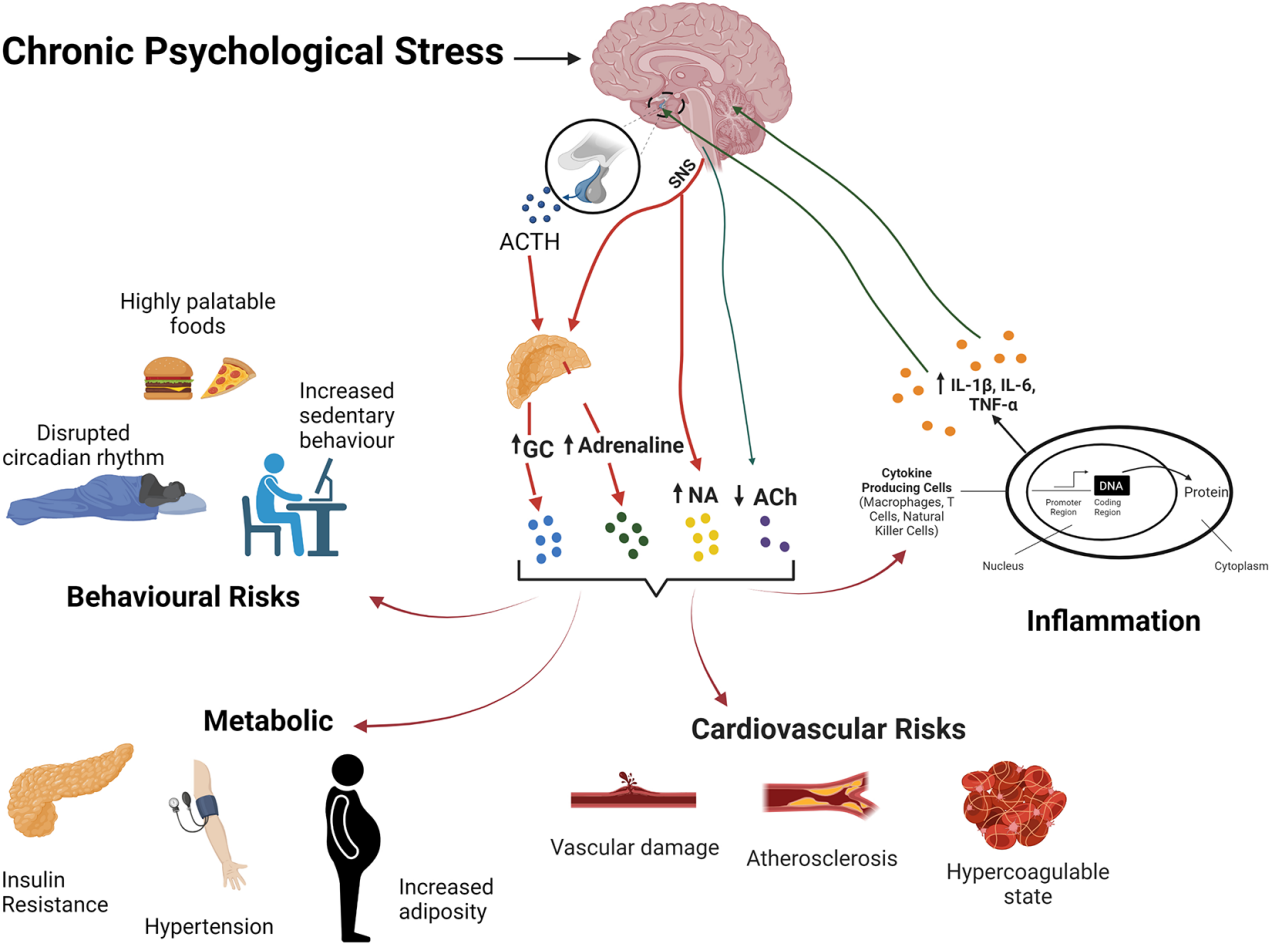
The stress response and its effects on body systems and CVD risk. Chronic stress activation of the HPA axis and SNS activity result in elevations in glvuocorticoids (GC) and catecholamines, and reduced PNS activity. Resultant behavoural/affective outcomes, immuo-inflammatory dysregulation, metabolic and cardiovascular effects collectively favour development of CVD.

Repetitive or chronic stress promotes disease *via* a broad disruption of homeostasis and adaptability (367, 368), incurring a significant biological cost or allostatic load (369). Shared biological networks may transduce chronic stress to multiple co-morbid disease states (6). Coronary artery disease is promoted by integrated influences of autonomic, neuroendocrine and immuno-inflammatory dysregulation on metabolism and lipid handling, thrombosis/haemostasis, and heart and vessel function and structure (Figure 2). Associated behavioural/affective impacts of chronic stress interact with these biological determinants to significantly increase risk of CAD, together with commonly co-morbid conditions including MDD and diabetes (6) (Figure 2). However, the stress response and its behavioural and cardiovascular influences are significantly sex-dependent (370). Indeed, fundamental adaptive mechanisms that provide resilience to stress differ between the sexes (371, 372).

#### 4.2.1. Stress drives behavioural risks

Chronic stress strongly predicts maladaptive coping or avoidance responses to a wide variety of provocations, including decreased social interactions, physical inactivity, altered sleep and dietary patterns (373, 374). These outcomes favour emergence of cardio-metabolic disorders. Behavioural drives of disease involve and are compounded by the influences of stress on neuroendocrine control, metabolic homeostasis and lipid handling, inflammation, and gut biology. Importantly, sex differences in coping strategies guide distinct behavioural responses to stress. The literature highlights a greater impact on eating and sleep patterns, together with immuno-inflammatory function in females vs. males.

The basis of sex-dependent behavioural responses to stress is complex. External psycho-social, cultural and life history factors all contribute to differences in stress-related behaviours and outcomes. Gonadal hormones play an important role (375), and sex differences in stress-related mood disorders emerge after puberty (376). Significant variability in gonadal hormone levels in women has also been implicated in differing incidence of stress-related disease (377). Androgens negatively regulate the HPA axis and reduce stress-dependent behavioural changes (378). In contrast, estrogen receptor (ER)α activity promotes HPA axis activity, increasing stress-dependent ACTH and corticosterone in male and female rodents (379, 380) and reducing negative feedback control of the axis (381). This is associated with anxiogenic influences of ERα activity (379, 382), although opposing behavioural effects of ERα activity are reported (383, 384), linked to reproductive history. Interestingly, anxiety-like behaviours are increased by ERα deletion in males (385) but not females (386). However, absence of these regulatory receptors over the life of knockout models complicates interpretation. Nonetheless, associations between ERα gene variants and anxiety and depressive disorders in humans (387–389) support the importance of sex hormones in the response to chronic stress.

Unfortunately, outcomes from animal studies, particularly in rodents, are equivocal and in some cases opposed to observations in humans. Studies in rats report reduced anxiety-like (390–392) and depression-like (393, 394) behaviours in females vs. males, suggesting greater female resilience. Others report no differences or increased anxiety- and depressive-like behaviours in female vs. male rats (395, 396). Similarly variable outcomes are evident in murine studies (397, 398), however there is evidence of greater biological or behavioural disruption in stressed female vs. male mice (399, 400), consistent with our own recent observations (12).

##### 4.2.1.1. Inflammation-dependent sickness behaviours

Inflammatory challenge induces “sickness behaviours” that closely mimic elements of stress dependent mood disorders, often cited evidence for key involvement of low-grade inflammation in the behavioural/affective impacts of chronic stress (401, 402). Interactions between inflammation and mood disorders (403, 404) have been highlighted in large population studies (405) and meta-analysis (406). A bi-directional interaction exists between inflammation and mood disorders, a positive feedback characteristic of stress-related disease (407, 408). Circulating cytokines communicate with the brain through both neural and humoral routes, influencing central control of mood and behaviour (409, 410). The biological mechanisms of cytokine-induced behavioural changes have been well studied in animal models, for example employing synthetic double-stranded RNA (poly I:C) or lipopolysaccharide (LPS) challenge to induce anxiety- (411) and depressive-like behaviour (412), respectively. Although chronic exposure may ultimately induce immune tolerance, prolonged changes in the brain and periphery emerge. For example, chronic LPS treatment induces sustained microglial activation (413), and persistent poly I:C exposure induces tumour necrosis factor (TNF)-α dependent neuroinflammation and alters expression of memory associated genes in frontal cortex (FC) and hippocampus (414).

The behavioural influences of inflammation are sex-dependent (177). Experimental evidence supports an enhanced immuno-inflammatory reactivity in women (415–417), although comparable outcomes in men and women have also been reported (418, 419). Studies in animal models provide support for greater neuroinflammatory reactivity in females vs. males (420). Distinct outcomes from *in vitro* vs. *in vivo* studies are relevant, confirming critical involvement of systemic (*in vivo*) mechanisms in the expression of sex differences. For example, higher female immunoreactivity is less evident in *ex vivo* models (421), with opposing evidence of greater reactivity in isolated immune cells from men *vs*. women (422, 423). This highlights the importance of assessing the complex biological influences of stress *in vivo* within an integrated systems biology framework (6).

The temporal pattern of behavioural response is also relevant in terms of sex differences. Engler et al. (181) assessed behavioural/affective effects of low level LPS challenge and reported similar mood, anxiety and fatigue levels in men and women over the initial 6 h of immune challenge, consistent with other reports (416, 424–426). However, sickness behaviours 24 h following LPS may be greater in women *vs*. men (427), suggesting a progressive emergence of sex dependent behavioural outcomes after the initial inflammatory response and peak cytokine changes. That said, there are also reports of greater depressive symptoms during the initial inflammatory response in women (93, 428).

##### 4.2.1.2. Dietary changes

Dietary patterns are changed with stress, in a manner generally promoting the development of CAD (429). This “emotional eating behaviour” (430) includes spontaneous binging and hyperphagia together with increased consumption of fatty foods, sweet foods and snacks (vs. decreased consumption of fruits and vegetables). It is well established that elevations in CRH and adrenaline with acute stress suppress appetite to induce a negative energy balance. Conversely, extended release of cortisol or corticosterone (with chronic stress) more generally increases appetite and motivation to eat (431) and modifies adiposity and fat distribution (432)—although not all studies agree (433). As with addictive drug behaviours, hyper-palatable food consumption with chronic stress is linked to increased mesolimbic dopaminergic system activity (434).

Unfortunately, few studies directly examine the importance of biological sex in determining stress-dependent eating patterns. Meta-analysis of links between stress and metabolic syndrome evidences a differing stress-metabolism relationship in females *vs*. males, suggestive of greater susceptibility of females to harmful stress-dependent behaviours (such as insomnia, physical inactivity and disordered eating) (435). Additionally, an analysis of relationships between BMI and post-traumatic stress disorder in Iraq and Afghanistan veterans identified sex effects, including preferential links between persistent obesity and PTSD in men vs. depression in women (implicating distinct sex-specific mediators of stress related weight gain). Women may experience more stress-dependent eating than men (436), and pre-clinical studies support sex-dependent patterns of stress feeding. For example, Anversa et al. (437)., investigated stress-induced binge-eating in food restricted and unrestricted mice, with results showing that males only displayed binge-like eating behaviour under food restricted conditions, whereas this behaviour was evident in *ad libitum*-fed females. This suggest potentially greater hedonic or reward behaviour in females, whereas male eating may be strongly dependent upon or reflects a homeostatic response to metabolic challenge. Nonetheless, opposing effects of stress are also reported, with chronic stress reducing food consumption in both mice (438) and rats (439), though dietary changes again appear more prominent in females. Temporal differences may be important: Pare et al. (439) found that food consumption was consistently reduced with increasing stress chronicity in females, whereas changes were transient (recovering to baseline) in male animals over the same period. Nonetheless, with only a 5-day duration of “chronic” stress in this study, outcomes may involve significant influences of acute stress.

##### 4.2.1.3. Reduced physical activity

Reductions in physical activity with stress favour development of metabolic and cardiovascular disorders. Increased physical activity, in turn, is cardioprotective and also anti-depressant. Indeed, physical fitness or aerobic capacity may be a dominant determinant of chronic disease risk (conversely health and longevity) (440). Levels of physical activity are reduced in chronic stress or stress-related mood disorders, explaining in part the link between stress/MDD and CVD (441). Similarly, chronic stress reduces running activity in mice (442). Anxiety sensitivity, a learned cognitive trait predisposing individuals to fearful misinterpretations of internal processes and sensations (443–445), is also associated with declining physical activity and fitness (446–448), together with cardiovascular risks including hypertension (449, 450), high cholesterol (451), atherosclerosis and arterial stiffening (452). A study by DeWolfe (453)., identified a significant, indirect effect of sex on physical activity in anxiety sensitivity (454), with female students reporting less activity and greater anxiety sensitivity than males. While Moshier et al. (373) found no sex differences in the AS-exercise relationship, it was noted that women have significantly less engagement with exercise than men (thus may be at greater risk of physical inactivity).

##### 4.2.1.4. Disrupted sleep patterns

Dysregulated sleep, including more frequent awakening and inability to fall into deep sleep, may be an important link between chronic stress and CVD (455). Under normal conditions, the HPA axis and SNS are suppressed in the initial stages of sleep, with activity increasing close to maximum circadian rhythm immediately after waking. The HPA axis and SNS influence the overall amount of rapid eye movement sleep (456), and chronic stress is associated with nyctohemeral activation of these systems, increasing the release of CRH, ACTH, cortisol/corticosterone, noradrenaline, and adrenaline (457), disrupting circadian clock genes in peripheral organs, and inducing nocturia, chronic insomnia and increased fatigue (458).

Excitatory orexins may play an important role in dysregulated sleep. Generated in the lateral hypothalamic regions, orexin neurons project to all brain regions. Of particular relevance, orexins influence regions associated with arousal, such as the locus coeruleus, to regulate responses to stressful stimuli (459). Effects of central orexin administration are well documented in animal models, supporting involvement in stress-mediated behaviour (460) and the dysregulation of HPA axis (461, 462) and SNS function (463, 464). Although it is clear acute stress up-regulates orexins, effects of repeated or chronic stress are less well defined: predictable (homotypic) chronic stressors generate conflicting outcomes (465–467), while unpredictable (heterotypic) chronic stressors up-regulate orexins (468). Lower orexin function may be indicative of stress resilience (469) or habituation (470). Examining sex-effects in rats, Grafe et al. found that females exhibit significantly less habituation to repeated restraint stress than do males, in association with higher levels of orexin neuronal activity (471).

#### 4.2.2. Stress disrupts metabolic homeostasis and lipid handling

Effects of chronic stress on neuroendocrine/autonomic function and inflammation interact to disturb whole body and organ specific metabolism. With the perception of stressful conditions, HPA axis and SNS activities and mediators increase energy supply *via* catabolic influences, liberating energy substrates such as glucose, amino acids, glycerol and fatty acids. However, prolonged activity with chronic stress can promote obesity, insulin-resistance, diabetes and metabolic syndrome. Psychosocial stress is a significant risk factor for excess weight gain (472) and obesity (473), associated with increasing adiposity (474), redistribution of fat tissue and accumulation of abdominal fat (475, 476). Exposure of healthy (non-obese) young men to long term stress increases abdominal obesity and dyslipidaemia (477). Chronic psychological stress in patients with stable ischaemic heart disease is similarly associated with higher body fat and detrimental lipid changes (478). Community-based studies also demonstrate a tendency for obese individuals to experience greater levels of stress-related disorders (479). Moreover, chronic stress may selectively promote particularly problematic visceral fat accumulation rather than subcutaneous fat, which is associated with low CAD risk and mortality (480). Mediators of stress-dependent metabolic dysfunction are noted below, together with potential sex effects.

##### 4.2.2.1. Glucocorticoid mediators

The link between glucocorticoids and metabolic disruption has been well established, exemplified in the increased weight gain, and visceral adiposity in Cushing's syndrome (481) and exogenous corticosteroid exposure (482). The link between glucocorticoid dysregulation and metabolic abnormalities is highlighted in mood disorders, with hypercortisolemic (and not normo-cortisolemic) depression associated with increased visceral fat (483). Increased glucocorticoid levels induce visceral fat accumulation through increases in dietary fat intake and hydrolysis of circulating triglycerides by lipoprotein lipase (484). Glucocorticoids also stimulate hepatic gluconeogenesis and inhibit glucose handling skeletal muscle and adipose tissue, promoting insulin-resistance (485, 486). There is evidence elevations in glucocorticoids contribute to insulin-resistance *via* increases in pancreatic islet proliferation and volume, insulin secretion capacity, and islet chaperone expression (487). Interestingly, despite anabolic effects on adipose and overall mass in humans, catabolic effects of glucocorticoids appear maintained in other tissues, with reductions in bone mineral density and lean body mass (488). These catabolic effects are more prominent in rodents vs. humans, with reductions of body weight in response to corticosterone intake (489) or repeated dexamethasone injections (490). However, adipose mass may nonetheless increase relative to overall body mass (489, 491).

##### 4.2.2.2. Altered SNS activity

The SNS is important in the integrated regulation of energy expenditure and intake to maintain long-term energy balance. Clinical and pre-clinical studies provide broad support for the involvement of sympathetic over-activity in development of obesity and metabolic syndrome (492), and stress-dependent metabolic dysregulation. Baseline sympathoadrenal activity has been shown to predict increases in body weight and development of insulin-resistance in an 18-year longitudinal study (493). Insulin-resistance and dyslipidaemia in a rat model of metabolic syndrome are worsened by chronic restraint stress, in association with increased noradrenaline levels and countered by β-adrenoceptor blockade (494). There is also evidence the greater effects of stress on LDL cholesterol and triglycerides in hypertensives vs. normotensives involve increased noradrenaline activity (495). Sympathetically mediated vasoconstriction may also functionally antagonize insulin-dependent glycaemic control *via* reductions in tissue blood flow, contributing to the development of insulin-resistance (496). There is evidence for an important interaction between SNS and NPY activities in development of obesity and metabolic disease. For example, Kuo and colleagues (497) report that stress dependent NPY release from sympathetic nerves promotes abdominal obesity and development of metabolic syndrome (including impaired glucose tolerance, hyperlipidaemia, hypertension, and increased concentrations of insulin, leptin and resistin). Outcomes in NPY over-expressing mice are also consistent with a role for altered SNS activity in NPY-dependent obesity and metabolic syndrome (498).

##### 4.2.2.3. Inflammation

Immuno-inflammatory activation in brain and periphery are a feature of chronic stress (499), with indirect effects on metabolism and regulatory systems throughout the body (500). Chronic low-grade inflammation induces insulin-resistance (together with endothelial dysfunction) (501) and may directly facilitate diet-induced obesity (502). A bi-directional positive feedback is evidenced in studies of dietary obesity in mice (503), with increased hypothalamic and hippocampal inflammation participating in a vicious feed-forward cycle of CNS dysfunction (504). This may involve increased blood brain barrier permeability, reactive glial cytokine production and circulating pro-inflammatory adipokines (505). In the periphery, members of the interleukin (IL)-1 family influence insulin-resistance and metabolic inflammation in obesity-associated disorders (506–508). Modulators of IL-1, NLRP6 and NLRP3 inflammasomes negatively regulate non-alcoholic fatty liver disease and steatohepatitis progression and contribute to aspects of metabolic syndrome (509). The inflammatory transcription factor NF*κ*B has emerged as an important metabolic regulator, with enhanced hepatic activity observed in high fat-fed mice (510, 511). As detailed further below, pro-inflammatory cytokines contribute significantly to vascular dysfunction and atherosclerotic disease (512).

##### 4.2.2.4. Lipid handling

Stress-dependent dyslipidemia similarly favours development of CAD. Chronic stress promotes an unfavourable pro-atherogenic lipid profile in humans (513) and animal models (514–517). This may include elevations in circulating cholesterol, low-density lipoproteins (LDLs), very-low density lipoproteins (VLDL) and triglycerides, and reductions in high-density lipoproteins (HDLs). Chronic mild stress in rats increases total, LDL and VLDL cholesterol together with triglyceride levels (and atherogenic index) without influencing HDL cholesterol (518). Increased sympathetic activity with stress, acting *via* β1, β2 or β3-adrenergic receptors in white adipose tissue, promotes lipolysis (519). Increased VLDL and decreased HDL levels, and facilitation of LDL entry into blood vessel walls, initiates and promotes the atherosclerotic process (520).

##### 4.2.2.5. Sex effects

Further work is needed to reveal the effects and importance of sex in stress-dependent metabolic dysregulation and disease. Nonetheless, there is evidence of sex specific changes in metabolism and lipid profiles. For example, animal studies suggest stress-induced changes in metabolic rate may be greater in females than males (521), which may reflect oestrogenic attenuation of sympathoadrenal and HPA responsiveness (522). The anabolic effects of glucocorticoids are more prominent in male compared with female mice, potentially involving oestrogenic protection and differing corticosteroid receptor expression/sensitivity in females (523). This may contribute to a greater relative risk of stress-dependent obesity and metabolic disturbance in females vs. males. It has been reported that chronic restraint stress reduces abdominal fat deposition in male rats while increasing fat in females (524). On the other hand, chronic social stress may induce weight loss specifically in female and not male mice (12).

Studies confirm sex differences in circulating lipid profiles under both stress and non-stressed conditions. Pre-menopausal females exhibit higher circulating HDL levels while males have higher triglyceride levels, with lipoprotein profiles converging after menopause (525). Reduced risk of CVD in premenopausal women has been attributed this higher concentration of HDL cholesterol (526). However, there is also evidence recurrent episodes of stress may eliminate this benefit in women (527). This agrees with evidence risk of stress-related disorders is differentially linked to lower HDL-cholesterol levels in women vs. lower LDL-cholesterol levels in men (528). Similarly, HDL-cholesterol is a more significant CVD risk factor in women, while LDL-C is more significant in men (529). Studies in rats indicate acute and chronic stress increase plasma cholesterol levels to a greater extent in ovariectomized females (compared with intact females or males), and that metabolic risk is influenced more by acute stress in males vs. chronic stress in females (530). Prenatal stress also exerts sex-specific effects on metabolism, although findings are mixed. This includes evidence of a female specific elevation in cholesterol levels, and a male specific fall in body weight in prenatally stressed mice (531), whereas others report a male specific increase in cholesterol and triglycerides (532).

#### 4.2.3. Chronic stress modifies vascular function and structure, and systemic blood pressure

Chronic stress induces endothelial dysfunction and detrimental vascular remodelling (533–535), effects that increase vascular resistance and favour local O_2_ supply:demand imbalances, hypertension, development of atherosclerosis, and acute coronary events. Stress-dependent endothelial dysfunction, linked to adverse cardiovascular outcomes in patients with CAD (362), arises with diverse stressors in both humans and animal models. For example, flow-mediated, endothelium-dependent vasodilation is impaired with chronic sleep deprivation and exam stress in healthy male college students (536), and with chronic carer stress in the elderly (537). The coronary microvascular impact of mental stress appears solely endothelium-dependent and is mirrored in peripheral vascular responses (538). Experimental studies support reduced nitric oxide (NO) bioavailability and vasodilatation (potentiating α-adrenoceptor-mediated vasoconstriction) in association with increased oxidative stress in animal models of chronic stress (518, 539–541). However, there is also evidence of reduced endothelium-dependent hyperpolarization-like relaxation in microvessels from rats subjected to chronic mild stress (542). Reduced endothelial-dependent dilation in arterial tissue from chronically stressed rats has also been linked to shifts in central (cortical) glutamate signalling (543), revealing a potential neurobiological mechanism of stress-dependent endothelial dysfunction. Intima and media hypertrophy arise in association with increased α-adrenoceptor vasoreactivity in rats exposed to chronic unpredictable stress (518). Arterial blood pressure also increases in models of chronic stress, in association with endothelial dysfunction, oxidative stress and increased angiotensin II levels (541). Causal involvement of RAAS activity is supported by beneficial effects of ramipril and losartan. Chronic stress may excessively activate the RAAS (544) and increase arterial sensitivity to angiotensin (545). Chronic elevations in angiotensin II also induce inflammation, endothelial dysfunction and senescence (546, 547), while angiotensin receptor antagonism protects against the effects of chronic stress (548). Sympathetic activity may indirectly promote endothelial dysfunction *via* its stimulatory effects on RAAS activity (549), together with inflammation and oxidative stress.

Glucocorticoids play a causal role in endothelial dysfunction, whereas involvement of sympatho-adrenergic activity is less clear and potentially indirect (533). Nonetheless, vasoconstrictor activity of catecholamines is consistently augmented with chronic stress, which likely contributes to hypertension and coronary abnormalities. Increases in cortisol inhibit endothelium-dependent vasodilation (550), reducing forearm blood flow responses to acetylcholine in healthy men for example (551). Cortisol directly reduces human endothelial eNOS expression (552), consistent with presence of a glucocorticoid response element in the eNOS promoter. Confirming the importance of glucocorticoids, effects of mental stress on flow-mediated vasodilation are negated by inhibition of cortisol production (350).

Involvement of the low-grade inflammation characteristic of chronic stress is indirectly supported by observations in humans and pre-clinical models. Cytokines reduce endothelial-dependent dilation in human veins *in vivo* (553), and acute inflammation with vaccine challenge in healthy volunteers is associated with pronounced endothelial dysfunction (554). As for cortisol, cytokines also down-regulate endothelial nitric oxide synthase (eNOS) in human coronary endothelium (555), and arterial endothelium of other species (556).

Chronic psycho-social stress promotes development of essential hypertension (496, 557–559), a major risk for CAD and infarction (560). Recent secondary analysis of the Isfahan Cohort Study (561), for example, indicates high stress levels increase the likelihood of hypertension by ∼40%, with significant links between hypertension and job conflict, job security, personal conflict, sexual and daily life in both sexes (independent of socioeconomic/lifestyle covariates), together with financial problems in males. Stress-dependent sympathetic activity promotes vessel remodelling and vasoconstriction (562), which with endothelial dysfunction and altered neuroendocrine and autonomic control, collectively favour development of hypertension (496, 585). Chronic stress impairs baroreceptor sensitivity and baroreflex function to increasing arterial pressure in humans (563, 564), while increased glucocorticoid levels are also associated with and increase the risk of hypertension (565, 566). Hypertension is additionally supported by associated neuroinflammation (567) and increases in renal renin and pituitary AVP secretion (568). Inhibition of neuroinflammation counters hypertension in different experimental models (569–572), and the hypertensive effects of angiotensin II/RAAS activity have also been linked to microglial activation and inflammatory cytokines within the PVN (573).

##### 4.2.3.1. Sex effects

The coronary and hypertensive influences of chronic stress are significantly dependent upon biological sex. Indeed, it is clear that coronary physiology and syndromes must be understood and managed in a targeted sex-specific manner (574–576). Importantly, women have greater microvascular density and baseline coronary perfusion (intermediate between healthy and CAD subjects) than men (577, 578), with less macro or obstructive CAD (579, 580). Evidence suggests women are more prone to coronary microvascular dysfunction than men (580, 581), including stress-dependent infarction (43, 44). Coronary microvascular dysfunction is also linked to sex-dependent Takotsubo cardiomyopathy (45) and heart failure with preserved ejection fraction (HFpEF) (582), both significantly more prevalent in women. Interestingly, recent work shows that a reduced coronary flow reserve in women is predicted by a blunted heart rate reserve, indicative of involvement of higher sympathetic activity in differing coronary outcomes (583). We recently reported that chronic social stress differentially increases coronary resistance in the hearts of female mice, while reducing resistance in males (12). Consistent with increased propensity to stress-dependent ischaemia in women (43, 44), the basis of this coronary dimorphism is unclear. However, female mice did exhibit greater inflammation than males, which impairs endothelial-dependent control in different vascular beds (553, 554).

Age and neuroendocrine changes are important interacting factors regarding sex effects on vascular and blood pressure control. For example, baroreflex sensitivity is higher in pre-menopausal women than either age-matched men or post-menopausal women (584). Oestrogen replacement therapy also increases baroreceptor sensitivity in postmenopausal women, attenuating onset and progression of cardiovascular disease and accelerating recovery from cardiovascular events (585, 586). Age-dependent elevations in sympathetic activity are more pronounced in women, while vagal tone and baroreflex sensitivity decline with age (587–589). Endothelial function falls progressively with age in men yet is relatively preserved in pre-menopausal women, before subsequently declining (590). This pattern is congruent with vasoprotection *via* gonadal hormones: endothelial function is reduced by prolonged gonadotropin-releasing hormone agonism (suppressing oestrogen generation) or acute gonadotropin-releasing hormone antagonism, and improved by hormone replacement in women (591); and endothelial function in young men is similarly impaired by aromatase inhibition to limit oestrogen generation (592). Stress-dependent increases in arterial pressure in ovariectomized rats are also reduced with hormone replacement, in association with increased vascular eNOS expression (593). Such observations support a protective role for oestrogen-sensitive vascular eNOS in limiting cardiovascular stress reactivity. Age dependent arterial stiffening is also more prominent in women, contributing to reduced baroreceptor firing and baroreflex sensitivity in older women vs. men (594). These age dependent reductions in baroreflex sensitivity may involve changes in peripheral afferent and/or efferent pathway control of the baroreflex system and sinus node function (594), with changes more dramatic in women compared to men (595).

Influences of testosterone on endothelial dysfunction are less clear: there is some evidence endothelial dysfunction is linked to declining testosterone levels (596, 597), although the opposite has also been reported (598). Meta-analysis indicates no significant associations between testosterone treatment and endothelial function, however studies are limited and outcomes mixed (599). There is also little research on the relationship between testosterone and endothelial function in women, however there is evidence of an association between low testosterone and endothelial dysfunction in post-menopausal oophorectomized women (600). Worboys et al. (601), also report that 6 weeks of testosterone supplementation in post-menopausal women receiving estradiol therapy improves both endothelial-dependent and -independent vasodilation. On the other hand, pre-clinical studies suggest potentially detrimental effects of testosterone on endothelial function (602), including inhibition of the vascular benefits of oestrogen (603). A higher blood pressure in male compared with female spontaneously hypertensive rats has been linked to testosterone sensitivity (and oestrogen insensitivity) of vasodilatory prostanoid generation, associated with sex differences in renal oxidative stress, heme oxygenase- 1 and arachidonic acid metabolism (604, 605). Consistent with a male propensity to stress-induced hypertension, chronic stress selectively increases arterial pressure in borderline hypertensive male but not female rats (606).

#### 4.2.4. Stress is pro-atherogenic

Chronic stress is strongly pro-atherogenic, reflecting integrated impacts of stress-dependent dyslipidaemia, vascular dysfunction/remodelling, thrombosis, hypertension, oxidative stress and low-grade inflammation, together with more direct atherogenic influences of SNS and HPA axis dysregulation. Chronic stress has been estimated to be a stronger independent risk factor for atherosclerosis (and intimal and media thickness) in an animal model of disease than total or LDL cholesterol (13). The broadly pro-inflammatory milieu induced by acute and chronic stress and SNS overactivity is pro-atherogenic (607), and the makeup, inflammatory profile and stability of atherosclerotic plaques is stress-sensitive. Chronic stress shifts plaques towards an unstable phenotype, with increased leukocyte and matrix metalloproteinase (608, 609) vs. reduced smooth muscle and collagen contents (634), and attrition of the fibrous cap (608). Plaque destabilisation in response to acute stress in ApoE^−/−^ mice (335) involves noradrenaline stimulation of endothelial adhesion molecule expression and chemokine release. Mast cells also participate in plaque destabilisation together with coronary vasospasm (610), are activated by chronic stress and corticotropin-releasing hormone, and are increased in CAD patients where they provoke acute coronary events (611–613). They have been shown to promote plaque destabilisation in animals subjected to acute stress (614), while chronic and early life stresses also increase mast cell numbers (615–617). However, involvement in chronic stress related atherogenesis awaits focussed study.

Sympathetic activity is an important mediator, with strong atherogenic potency of catecholamines (618, 619) readily demonstrable in animal studies (620, 621), even in the absence of altered blood pressure and cholesterol levels (619). Increased blood pressure reactivity to mental stress, governed by SNS activity, is also positively associated with development of atherosclerosis (622). Sympathetic activity stimulates splenic hematopoietic progenitor cell proliferation and myeloid cell development (623), and leukocytes express a more pro-inflammatory transcriptional profile (624), with increased expression of C-reactive protein, IL-1, IL-6 and TNF (625). Ablation of SNS activity with 6-hydroxydopamine reportedly counters the up-regulation of endothelial adhesion molecules in models of AMI (626).

Glucocorticoids are also pro-atherogenic (627). Pre-clinical studies confirm pro-atherosclerotic effects of cortisol/corticosterone in different models (628, 629). Priming by prior exposure to either glucocorticoids or noradrenaline also sensitises monocytes/macrophages to subsequent inflammatory challenge (630), effectively amplifying the effects of repeated stress. Stress and SNS-dependent RAAS activation additionally promotes atherosclerosis (656, 657). Angiotensin II activation of type 1 receptors increases leukocyte adhesion molecule and inflammatory mediator expression in endothelial cells (631), which possess glucocorticoid receptors that render them directly sensitive to HPA axis activity (632). Increased arteriolar leukocyte adhesion in response to angiotensin-II has been linked to TNF-α dependent signalling (633). Experimental studies also support involvement of pro-inflammatory toll-like receptor 4 and nuclear factor-kappaB signalling in the atherogenic effects of stress in ApoE^−/−^ mice (634), and a role for oxidative stress (and hyperlipidaemia) in stress related atherosclerosis (662). Endothelial dysfunction and abnormal NO signalling, reflecting in part the influences of inflammation and oxidative stress, also play an important role (663). Altered reverse cholesterol transport (RCT) could also participate, though this has received relatively little research attention. There is evidence chronic stress augments the inhibitory influences of high-fat feeding on RCT (635), although these investigators found no influence of stress alone on markers of RCT.

##### 4.2.4.1. Sex effects

There is evidence of significant influences of sex on stress related atherogenesis (Table 1), however focussed research is needed. For example, Chumaeva et al. report that, in the context of the syndrome of vital exhaustion (characterized by fatigue and irritability), only men with reduced arterial elasticity show an increased risk of atherosclerosis in early life, with no such risk in women with either high or low arterial compliance (636). While inconclusive, the authors suggest this may reflect better coping with stressful risk factors in women than men. Shivpuri et al. (637), also found evidence of sex-dependent influences of stress on inflammation in the Multi-ethnic Study of Atherosclerosis. Experimental studies also identify sex-dependent influences of early life stress on inflammation and mast cell activity: early weaning stress increases mast cell number and activity in pigs to a significantly greater degree in females vs. males (616), consistent with evidence repeated maternal separation stress selectively increases hippocampal mast cell numbers in females and not males (617). Such observations implicate mast cells as an understudied mediator of the sex-specific effects of early life adversity.

#### 4.2.5. Stress is pro-thrombotic

Thrombosis links chronic stress, mood disorders and CAD (638). Increased platelet–leukocyte aggregates are evident in CAD patients, and platelet activity is increased by high level stress and in people prone to stress-related depression (639). Mental stress induces prolonged elevations in pro-inflammatory platelet activity (321), and mental stress induced ischaemia is associated with increased platelet aggregation (640). Animal models similarly evidence increased thrombosis in response to chronic stress (641, 642). Increases in thrombopoiesis and platelet activity with stress involve both glucocorticoid and sympathetic influences (and associated inflammatory/ROS responses), together with potential roles for serotonin (643) and brain-derived neurotrophic factor (BDNF) signalling (204, 644). The sympathoadrenal activation with chronic forms of stress primarily enhances coagulation, while fibrinolysis may be additionally influenced by acute stressors (322, 645, 646). This may reflect a pro-survival response aimed at limiting blood loss in threatening fight-or-flight settings. However, such effects can in the longer term promote CAD and risk of acute coronary events (647, 648).

Platelet levels, activity and aggregation are all increased with glucocorticoid administration (332, 333), and under conditions of chronically elevated cortisol (649). Increased thrombin-induced platelet aggregation with high work stress is linked to elevations in cortisol (650). There is also experimental evidence ACTH promotes arterial thrombosis, with ACTH (yet not cortisol) acutely amplifying agonist-induced platelet aggregation (651). Associated SNS activity is also involved in stress-axis dependent thrombosis, with animal studies confirming key involvement in the pro-coagulant effects of chronic stress (652). Exposure to either stress or catecholamine increases thrombopoiesis, platelet reactivity and GPIb, GPIIb-IIIa complex and P-selectin expression (322, 334, 646, 653) in an α2-adrenoceptor dependent manner. *In vitro* studies confirm that adrenaline increases platelet activation/aggregation and clot formation *via* α2a-adrenoceptor activity (654).

Serotonergic signalling may also participate in stress-dependent platelet activity and thrombosis. Serotonin mediated platelet activation is enhanced in people with stress-related MDD (655), and an early review of the literature (688) highlighted varied evidence of hyperactive platelet serotonin receptor signalling in depressive patients which may increase risk of thromboembolic events. Subsequent studies confirm increased platelet serotonin receptor signalling and reactivity in response to acute or chronic stress (640, 656). Recent work also shows that depression is associated with higher serotonin receptor density in CAD patients, with increased platelet serotonin reactivity in depressed patients with minor adverse cardiac events (657).

Signalling by the neurotrophin BDNF, and specifically a BDNF^Val66met^ polymorphism, may additionally link platelet function to stress and MDD (204). Chronic stress disrupts BDNF signalling, which is considered an important determinant of stress resilience and mental health outcomes (658). Recent work shows that BDNF^Val66Met^ mice have increased propensity to thrombosis, with sub-chronic stress (7 day restraint stress) inducing a pro-thrombotic phenotype (659). This includes increased leukocyte and platelet numbers, heightened platelet responses, and increased platelet/leukocyte aggregates, P-selectin and GPIIbIIIa expression, and arterial tissue factor activity. More recent experimental evidence supports involvement of increased platelet α2-adrenoceptor signalling: the pro-thrombotic phenotype in BDNF^Met/Met^ mice is linked to platelet over-expression of α2A-adrenoceptors, involves noradrenaline and is rescued by select α2A-adrenoceptor antagonism; and platelets from homozygous BDNF^Met/Met^ CAD patients are similarly over-reactive and over-express α2A-adrenoceptors (205).

Additional pro-thrombotic influences of chronic stress include increased reactive oxygen species (ROS) and nicotinamide adenine dinucleotide phosphate (NADPH) oxidase dependent platelet activity (660), and dipeptidyl peptidase-4 expression (694), which may promote both oxidative stress and von Willebrand factor cleavage by ADAMTS13). However, stress-dependent elevations in dipeptidyl peptidase-4 may accelerate atherosclerosis *via* a multiplicity of mechanisms (661).

##### 4.2.5.1. Sex effects

The importance of sex on the association between stress related disease and platelet function has been somewhat neglected. A preliminary analysis of sex effects in the platelet-MDD association by Izzi and colleagues (662) evidences sex-dependent links between depressive symptoms and both platelet volume and variability in size. In a cohort of patients with stable IHD, Samad et al. found evidence of increased platelet sensitivities to both serotonin and adrenaline in women compared with men, together with greater mental stress induced ischaemia and collagen-stimulated platelet aggregation (640) (Table 1).

#### 4.2.6. Stress modifies myocardial control and promotes hypertrophy and failure

Just as vascular control and structure are altered, myocardial function and makeup are significantly modified with chronic stress. Autonomic dysfunction and a shift towards sympathetic vs. parasympathetic activity underlie a hallmark of chronic stress and associated depression—reduced heart rate variability (HRV). These autonomic changes are responsible for impaired baroreflex control of cardiac function (and blood pressure) (563). Animal studies link susceptibility to chronic stress to increased sympathetic tone and impaired baroreflex control (663). Angiotensin II signalling may play some role in these autonomic and baroreflex responses to chronic stress (664). Increased sympathetic tone and impaired baroreflex control, in turn, favours coronary and cardiac dysfunction, hypertension and cardiac hypertrophy. This is consistent with evidence a low HRV predicts the onset of (665) [and falls further with (666)] hypertension, and a relationship between low HRV and left ventricular hypertrophy (667, 668), although the latter may vary significantly with race (669). A reduced HRV significantly increases the risk of a cardiac event: analysis of Framingham Heart Study data reveals a low HRV increases risk of an acute cardiac event over a mean follow-up of 3.5 years by ∼1.5 fold (670). Reductions in HRV following AMI or CABG are also linked to worsened outcomes (671, 672). However, despite generally reflecting the balance of sympathetic and parasympathetic influences in cardiovascular control, it should be noted that the mechanistic basis of the relationship between HRV and coronary or myocardial disease remains to be firmly established, and may well be indirect—changes in HRV may serve as a secondary biomarker of chronic stress, which influences CAD risk and outcomes *via* diverse mechanisms parallel to SNS activity.

Together with altered cardiovascular control, prolonged sympathetic activity and associated inflammation, and shifts in adipokine and other endocrine signalling, promote cardiac hypertrophy and failure. Hypertrophy renders the myocardium susceptible to O_2_ supply:demand imbalance and is a risk factor for CAD development and cardiovascular morbidity and mortality. Chronic stress also exacerbates pressure overload dependent heart failure in animal models, in association with shifts in cardiac apoptosis and fibrosis (673). The relationships between low HRV and left ventricular mass (667, 668) is consistent with involvement of autonomic imbalance in hypertrophy. Supporting causal involvement, recent experimental work shows that reinstating HRV reverses detrimental remodelling in an ovine model of heart failure (674).

Altered adipokine signalling may also play some role. Adiponectin is cardioprotective, limiting hypertrophy and heart failure (675, 676) and protecting against sympathetic and myocardial remodelling after infarction (284). Adiponectin levels are generally repressed with chronic stress (283), potentially facilitating stress-dependent dysfunction and hypertrophy. However, early life stress is reported to specifically increase adiponectin levels in female and not male rats (192), reflecting a potentially protective sex dependent response. Interestingly, adiponectin dependent signalling has also been linked to fear extinction, with reduced adiponectin associated with emergence of post-traumatic stress disorder (285, 677). Leptin signalling is also modified with chronic stress, including elevated circulating levels linked to evolving leptin resistance. However, while leptin can be protective, for example limiting hypertrophy and apoptosis, increased levels are strongly linked to CAD risk and poor outcomes (675, 676). Increased leptin levels with stress may thus predispose to CAD development, mortality and morbidity. Early life stress may induce sex dependent changes in leptin, including evidence of a selective fall in leptin in male and not female rats (678). However, the roles of leptin in stress-dependent disease remain unclear (675, 676, 679) and require further study, particularly in terms of the effects of sex.

##### 4.2.6.1. Sex effects

Oestrogen may protect against cardiac hypertrophy/failure, while post-menopausal women may become sensitised to these outcomes. The protective effects of oestrogen in CAD and maladaptive hypertrophy may involve multiple mechanisms, including influences on sympathetic activity and endothelium-dependent and -independent control of microvascular function (680), lipid handling and profiles, and key transcriptional regulators such as the nuclear factor of activated T-cells (NFATs) (681). Experimental studies show oestrogen does protect against cardiovascular dysfunction in models of Takotsubo syndrome (682, 683), and counters cardiomyopathy in female rodents exposed to chronic catecholamine stress (682). Others report that oestrogenic protection against stress-dependent cardiomyopathy may involve increased β_2_AR–G_αs_ signalling activity and reduced catecholamine levels (684).

Both HFpEF and Takotsubo cardiomyopathy are of interest in terms of sex-dependent CVD and stress. Increased immuno-inflammatory reactivity to stress in women is congruent with proposed involvement of coronary vascular inflammation and dysfunction in female linked HFpEF (47, 582). Similarly, Takotsubo cardiomyopathy is thought to involve excessive sympathetic drive (45, 685), and its dominance in post-menopausal women is consistent with enhanced stress-dependent disruption of autonomic control in females as a result of reduced oestrogenic protection (685).

Women are 4- to 10-fold more likely to suffer Takotsubo than men (46), although there is evidence males may suffer greater in-hospital complications (686). The most well-established mechanism in Takotsubo cardiomyopathy is sympathetic over-activation, which involves increased noradrenaline release (and potentially changes in myocardial sensitivity) (45, 685). The effects of stress on the LC-noradrenaline axis, which enhances HPA activity and release of adrenal adrenaline/noradrenaline into the circulation, are sex-dependent (101). Locally released catecholamines may also participate, and may be more damaging than those transported in the circulation (687). Increased myocardial sensitivity to catecholamines in females—particularly within the left ventricular apex—could underlie apical changes characteristic of Takotsubo syndrome (364). Coronary vascular dysfunction may also contribute, with reductions in apical perfusion documented (688). Sympathetic activity favours coronary dysfunction (689), and associated inflammation (690) contributes to endothelial damage. Release of vasoconstrictors and related ROS generation may also participate in coronary vascular changes (691). Thus, both the stress-related sympathetic activity and coronary dysfunction implicated in Takotsubo cardiomyopathy are sex-dependent. This is congruent with the strong sex-dependence of stress related SCAD, also potentially involving excess sympathetic activation (30, 31). Mitochondrial dysfunction may in turn be exacerbated by these autonomic and vascular changes, and there is evidence of an impaired myocardial energy state in Takotsubo cardiomyopathy (692). Though inflammation is also implicated (693, 694), it is interesting to note that that the limited information available on sex differences in the disorder—largely from a series of studies in Japanese populations—supports lower C-reactive protein and leukocyte levels in female patients (46). An extremely limited comparison of cardiac gene expression in a single male patient (>70 years of age) and single female patient (>80 years of age) suggests greater changes in mitochondria related genes in the male compared with greater ECM-receptor and -integrin interaction genes in the female patient (695). However, whether such a difference might be causal, and robust across the sexes, remains to be tested. Such findings nonetheless further evidence of distinct disease processes in males and females.

#### 4.2.7. Other pathophysiological influences of chronic stress

Stress increases myocardial vulnerability to arrhythmias and coronary ischaemia (696–700). Studies in mice also suggest that mild chronic stress may interact synergistically with a western diet to worsen myocardial ischaemic injury, together with metabolic homeostasis and anxiety-like behaviour (701). We more recently report that chronic modes of social or unpredictable mild stress worsen myocardial ischemic tolerance in male mice, with cardiac outcomes correlating with circulating noradrenaline and leptin levels, and cortical and hippocampal expression of monoamine and inflammatory genes (702). However, we also report that chronic social stress reduces myocardial ischaemic tolerance in male and not female mice, in associated with a sex-specific decline in ventricular expression of genes involved in energy metabolism, mitochondrial biogenesis and cardioprotection (12). While still poorly studied (703), there is also evidence chronic stress inhibits cardioprotective survival kinase activation (704). Our unpublished findings indicate that chronic restraint stress in mice prevents the protective phospho-activation of myocardial protein kinase B (AKT) in response to preconditioning stimuli. Information on the sex dependence of these cardiac effects is lacking.

Mitochondria are responsive to stress (705), and dysfunction is linked to CVD, MDD and chronic stress (706–710). Mitochondrial dysfunction may be a common link between chronic stress and multiple diseases (711). Chronic stress inhibits mitochondrial respiration in nervous tissue of animal models (712), with a link between duration of stress and degree of inhibition (713). Stress effects involve in part direct glucocorticoid responses, with mitochondria possessing glucocorticoid receptors together with sex hormone receptors/transcription factors, providing sensitivity to endocrine function and sex (714, 715). While the sex dependence of mitochondrial dysfunction in CAD awaits detailed analysis, sex does influence cardiac mitochondrial responses to stress (705) and sex differences are evident in mitochondrial roles in stress-related mental disorders (716). Experimental studies also demonstrate broad “mito-protective” functions of oestrogens (717–719), which directly enhance mitochondrial oxidative phosphorylation (720, 721). Apparent resilience of female cardiac mitochondria to acute stressors has been linked to direct effects of oestrogen (705). Others link improved ischaemic tolerance in female mouse hearts to oestrogen dependent improvements in mitochondrial connexin-43 content and phosphorylation (722). There is evidence mitochondrial dysfunction (in brain tissue) is more sensitive to oestrogen than testosterone deficiency (723). While cardiac mitochondrial dysfunction is enhanced by both testosterone (724) or oestrogen deficiency (725) in insulin-resistant male and female rats, there is evidence sex hormone deprivation is more likely to induce cardiac mitochondrial dysfunction in healthy and insulin-resistant male vs. female mice (726). Pereira et al. (727), recently found that reduced maternal nutrition in a baboon model dysregulates foetal cardiac mitochondria in a sex specific manner, which could contribute to sex-dependent programming of adult cardiac dysfunction. Similarly, Louwagie et al. found that foetal exposure to maternal glucolipotoxicity modifies metabolism, propensity to cell death and risk of adult heart disease *via* mitochondrial mechanisms in a sex-dependent manner (suggestive of improved mitochondrial quality control in females, and greater vulnerability in adult males) (728).

## 5. Conclusions

Differing responses to chronic forms of psychosocial stress contribute to sex-dependent CAD and its impacts, however significant knowledge gaps exist in terms of the interactions between sex, stress and disease. This demands an increased focus on sex biology and dependent disease mechanisms. Evolutionary, socio-cultural and intrinsic biological factors all interact in generating differing perceptions of stress, and the biological transduction of stress to different behavioural, emotional and physiological outcomes. This distinct stress biology involves molecular, structural and functional differences within the HPA axis and ANS, together with immuno-inflammatory, neurotrophin and endocrine function, pyrimidine metabolism and sirtuin signalling, among other players. Baseline myocardial and coronary physiological differences may interact with these distinct stress responses in the sex-dependent development of cardiovascular disorders, particularly those linked to coronary vascular (dys)function. Despite these clear biological differences, the daily experience of stress remains biased by socio-cultural and economic factors, and stress axis reactivity and responses are similarly influenced by such external determinants. Significant advances in the prevention of chronic disease in women can thus be made at fundamental socio-economic levels. At the same time, greater understanding of sex and stress biology can better inform approaches to CAD prevention (targeting sex specific risk profiles) and therapy (targeting sex dependent biological mechanisms) in both men and women.
